# Palliative care utilization in oncology and hemato-oncology: a systematic review of cognitive barriers and facilitators from the perspective of healthcare professionals, adult patients, and their families

**DOI:** 10.1186/s12904-020-00556-7

**Published:** 2020-04-13

**Authors:** Marco Bennardi, Nicola Diviani, Claudia Gamondi, Georg Stüssi, Piercarlo Saletti, Ivan Cinesi, Sara Rubinelli

**Affiliations:** 1 Swiss Paraplegic Research, Person-centered Healthcare & Health Communication; University of Lucerne, Department of Health Sciences and Medicine, Guido A. Zäch Strasse 4, 6207 Nottwil, Switzerland; 2 Oncology Institute of Southern Switzerland, Palliative Care, Ospedale San Giovanni, 6500 Bellinzona, Switzerland; 3 Oncology Institute of Southern Switzerland, Hematology, Ospedale San Giovanni, 6500 Bellinzona, Switzerland; 4 Oncology Institute of Southern Switzerland, Medical Oncology, Ospedale Regionale Lugano, 6962 Viganello, Switzerland; 5 Palliative TI – Associazione Cure Palliative Ticino, Via San Leonardo, 6599 Cadenazzo, Switzerland

**Keywords:** Cancer, Hematologic neoplasms, Cognitive barriers, Cognitive facilitators, Palliative care

## Abstract

**Background:**

Despite the high potential to improve the quality of life of patients and families, palliative care services face significant obstacles to their use. In countries with high-resource health systems, the nonfinancial and nonstructural obstacles to palliative care services are particularly prominent. These are the cognitive barriers -knowledge and communication barriers- to the use of palliative care. To date no systematic review has given the deserved attention to the cognitive barriers and facilitators to palliative care services utilization.

This study aims to synthesize knowledge on cognitive barriers and facilitators to palliative care use in oncology and hemato-oncology from the experiences of health professionals, patients, and their families.

**Methods:**

A systematic review was conducted. PubMed, PsycINFO, International Association for Hospice and Palliative Care/Cumulative Index of Nursing and Allied Health Literature (IAHPC/CINAHL), and Communication & Mass Media Complete (CMMC) were systematically searched for the main core concepts: palliative care, barriers, facilitators, perspectives, points of view, and related terms and synonyms. After screening of titles, abstracts, and full-texts, 52 studies were included in the qualitative thematic analysis.

**Results:**

Four themes were identified: awareness of palliative care, collaboration and communication in palliative care-related settings, attitudes and beliefs towards palliative care, and emotions involved in disease pathways. The results showed that cognitive barriers and facilitators are involved in the educational, social, emotional, and cultural dimensions of palliative care provision and utilization. In particular, these barriers and facilitators exist both at the healthcare professional level (e.g. a barrier is lack of understanding of palliative care applicability, and a facilitator is strategic visibility of the palliative care team in patient floors and hospital-wide events) and at the patient and families level (e.g. a barrier is having misconceptions about palliative care, and a facilitator is patients’ openness to their own needs).

**Conclusions:**

To optimize palliative care services utilization, awareness of palliative care, and healthcare professionals’ communication and emotion management skills should be enhanced. Additionally, a cultural shift, concerning attitudes and beliefs towards palliative care, should be encouraged.

## Background

The relevance of palliative care services and their integration into the traditional medical model is widely recognized. In 2014, the World Health Assembly urged countries to integrate palliative care into their health care systems [1]. This goal has not yet been achieved, as palliative care services are not available, due to several various reasons, to all those patients experiencing a serious chronic disease in most countries, even in high-resource systems [2, 3].

According to the *Health Care Access Barriers Model* [4], barriers to health services use include *Financial barriers*, that is cost of care and health insurance status barrier, *Structural barriers*, namely institutional and organizational barriers, and *Cognitive barriers*, including knowledge and communication barriers. The three categories of barriers affect health care utilization individually and in concert, and are associated with late presentation to care, and lack of treatment, which translates in poor health outcomes [4].

Particularly in developed countries, where palliative care services are part of the healthcare system [3], the nonfinancial and nonstructural barriers and facilitators to palliative care use highly affect the utilization of palliative care for patients and their families. Additionally, even in developing countries, where resources are limited, cognitive barriers are prominent, as financial choices and organizational decisions are affected by cognitive aspects of the type of services in which to invest both at the system and the organizational levels [3]. Moreover, cognitive barriers are directly modifiable through targeted strategies.

In the specific case of palliative care services, referrer reluctance as well as patients and families reluctance are related to cognitive barriers, such as lack of understanding of the benefits of referral or avoidance of talking about end of life and death with the belief that this will allow avoidance of death itself [3].

Systematic literature reviews have synthesized research on barriers and facilitators to palliative care from a general perspective, without going into depth on cognitive barriers. Potential barriers include patients’ and doctors’ (e.g., general practitioners [GPs]’) ambivalence about discussing ‘bad news’ of a diagnosis [5], which is relevant as it may affect the GP-patient relationship. Other barriers include lack of human resources, financial constraints, limited infrastructure for palliative care [6], and lack of standardized referral criteria for palliative care services [7]. Potential facilitators for palliative care use include the availability of relevant specialized secondary services in integrated palliative care, which were shown to be highly valuable both for the hospital (lower costs) and for the patient (better use to certain palliative care services including improving functional status) [8]. In addition, these studies found that stakeholder engagement, financial support, a supportive learning environment for HPs, and community networks might facilitate palliative care use [6], as well as appropriate communication styles, perceptions of patient readiness [9], knowledge of palliative care [7, 9], and education on palliative care among HPs [7].

In particular, this review aims at identifying the cognitive barriers and facilitators to the use of palliative care, and at giving to these a deserved attention including a deeper exploration compared to previous broad systematic reviews. More specifically, we aim at analyzing perceived cognitive barriers and facilitators, to the utilization of palliative care, from the perspective of healthcare professionals, oncology patients and their families. The broad goal of this review is to identify targets to overcome the obstacles and encourage the enablers in order to reinforce health care systems towards an optimal utilization of palliative care.

## Methods

### Design

A systematic review was conducted following established guidelines to ensure rigour and transparency [10]. The Preferred Reporting Items for Systematic Reviews and Meta-Analysis: PRISMA statement [11] was followed to standardize the reporting. Qualitative, quantitative and mixed-method studies were included. A textual narrative synthesis [12] approach was adopted to present the results, more specifically, this review main goal was to explore diverse types of studies with no scope to explore relationships within and between studies. This approach included thematic analysis, used to extract the main themes [13], and tabulation in order to analyze the findings. Barriers and facilitators pertaining to areas other than personal and relational aspects, such as institutional and organizational barriers and facilitators, were not included in the analysis.

### Identification of relevant literature

Four databases (PubMed, PsycINFO, International Association for Hospice and Palliative Care/ Cumulative Index of Nursing and Allied Health Literature [IAHPC/CINAHL], and Communication & Mass Media Complete [CMMC]) were searched in June 2019. Search terms covered the concepts of palliative care and barriers and facilitators to referral and use (Additional file 1). Terms to cover palliative care in the included ‘palliative’, ‘supportive’, ‘end of life’, ‘terminal’, and ‘hospice’, which were searched with ‘care’ or ‘medicine’ or ‘treatment’ or ‘therapy’, as these have been used with similar meanings internationally. For each study, we checked that the type of care described matched with the concept of palliative care adopted here [14] and was relevant to the research question. Other terms included were ‘facilitator’ and ‘barrier’ (and synonyms) and ‘attitude’, ‘point of view’, ‘perspective’, ‘angle’, ‘position’, ‘thought’, ‘belief’, and ‘idea’ (for details see Additional file 1). Keywords were generated by examining other review papers in the palliative care literature and using an English thesaurus. Keywords were combined with standard MeSH terms from PubMed and subject headings for the other databases. Moreover, the definitions of barriers and facilitators used were the following: barriers are actual or perceived factors such as a perception or belief that make it difficult or impossible for patients to access palliative care; facilitators are actual or perceived factors as a perception or belief that make it easier or more likely for patients to access palliative care.

### Eligible studies

Papers focusing on palliative care and oncology or hemato-oncology were included. Inclusion criteria were the following:

- Study types: peer-reviewed studies presenting original qualitative, quantitative, and mixed-method studies with an exploratory descriptive approach. Systematic reviews, commentaries, editorials, newspaper articles, and other forms of popular media were excluded.
- Population or participants: papers from all over the world were examined. Studies including HPs, patients with cancer and families were eligible. Studies including patients with disease other than cancer were excluded. This review was limited to studies on adults, since perceived barriers and facilitators to use pediatric palliative care differ from those to use adult palliative care [15, 16].
- Setting: oncology, hemato-oncology, and general practice which includes oncology/hemato-oncology patients.
- Outcome measures: primary outcomes of interest were palliative care use, access, provision, implementation, and integration. Studies with diverse perspectives were included (e.g., hemato-oncologists’ and GPs’ perspectives). Studies that included palliative care as a range of services provided by a multidisciplinary team were examined; studies focusing on a single aspect of palliative care services (e.g., spiritual care only) were not considered.
- Language: studies in English only were included.

There is no consistency in defining palliative care in the literature [17, 18]. We refer to the WHO definition: “Palliative care is an approach that improves the quality of life of patients and their families facing the problem associated with life-threatening illness, through the prevention and relief of suffering by means of early identification and impeccable assessment and treatment of pain and other problems, physical, psychosocial and spiritual” [14]. Studies were included if they explicitly specified that the patients or health professionals were receiving, providing, or referring to palliative care services. Some studies referring to hospice care (or other terms, see *Identification of relevant literature)* that also investigated palliative care provision were considered, but only those in which palliative care was specifically addressed were finally included in the review.

### Data extraction and data synthesis

Included studies were heterogeneous, not allowing meta-analysis of quantitative studies or meta-synthesis of qualitative studies. A textual narrative synthesis, which aims to describe studies, by arranging these into homogeneous groups, through thematic analysis, and compares similarities and differences across studies [12] was conducted. The textual narrative synthesis of the results [12, 19] was performed by extracting relevant characteristics from the selected studies, charting them in tables (Tables 2, and Additional file 2: Table 3), and producing a textual summary of the results. Each study was coded using an ad-hoc data extraction sheet according to the following characteristics: Author name, year of publication, aim, design, participants (number and type), country, data collection method and sample, barriers or facilitators to palliative care services use, strategies, and outcome(s). Extracted barriers and facilitators were tabulated and synthesized thematically through a thematic analysis. The thematic analysis was used to identify and develop themes across studies, by using line-by-line coding, developing descriptive themes, and generating analytical themes [12]. Emergent themes are included in Table 1, and Additional file 2, Table 3. The criteria for the inclusion of barriers and facilitators in the relevant themes are reported in the paragraph “Thematic Analysis” in the results section. Regular debriefings and discussion of the themes among the three reviewers (M.B., N.D. and S.R.) and discussions with the other co-authors (C.G., P.S., I.C., and G.S.) were used to validate the findings.

**Table 1:** Themes of perceived cognitive barriers and facilitators to palliative care use

Themes	Examples of barriers	Examples of facilitators
**Theme 1: Awareness of palliative care**		
Awareness, knowledge, education or experience among health care professionals	Lacking understanding of the broad applicability of PC among HPs [51].	Improving education on PC for HPs in general, including experiencing observation and receiving support [39].
Awareness among patients and families	Patient and families do not know the purpose of PC involvement [64].	Increase of opportunities of patient education on PC for instance videos [64].
**Theme 2: Collaboration and communication in health care settings**		
Collaboration and communication between health care professionals and patients and their families	Health care professionals’ difficulty in communicating patients’ prognoses [54].	The use of short assessment scales, communication note-books and medical files with non-physical information [23].
Collaboration and communication between health care professionals	Difficulty in dealing with conflicting information about the goals of care which are them given among nurses [26].	PC team strategic visibility in patient floors and hospital-wide events and PC team unintentional visibility like being present around the hospital [64].
**Theme 3: Emotions involved in disease paths**		
Emotions among health care professionals	Barriers to discuss PC: emotional bond, emotional discomfort among oncologists [34].	Further education and training focused on dealing properly with patient psychosocial and emotional problems for HPs [52].
Emotions among patients and their families	Emotions in patients such as sadness/suppression, unrealistic expectations, infinite trust and faith in medicine, giving up hope, overly optimistic about life expectation [34].	Assessing psychological condition and treating mental disorders of patient [65].
**Theme 4: Attitudes and beliefs towards palliative care**		
Attitudes and beliefs among health care professionals	Belief that PC is not appropriate for those who have complex problems without physical symptoms among HPs [38].	Provision of clear, convincing, scientific support, concerning palliative care, for recommendations from the PC team [64].
Attitudes and beliefs among patients and their families	Interpretation of earlier and broader PC consultations as a cost saving measure (rather than clinically beneficial measure) among patients and their families [58].	Renaming ‘palliative care’ to ‘supportive care’, as patients were more receptive to the second name [57].

## Results

### Identification and selection of the literature

**Fig. 1:**
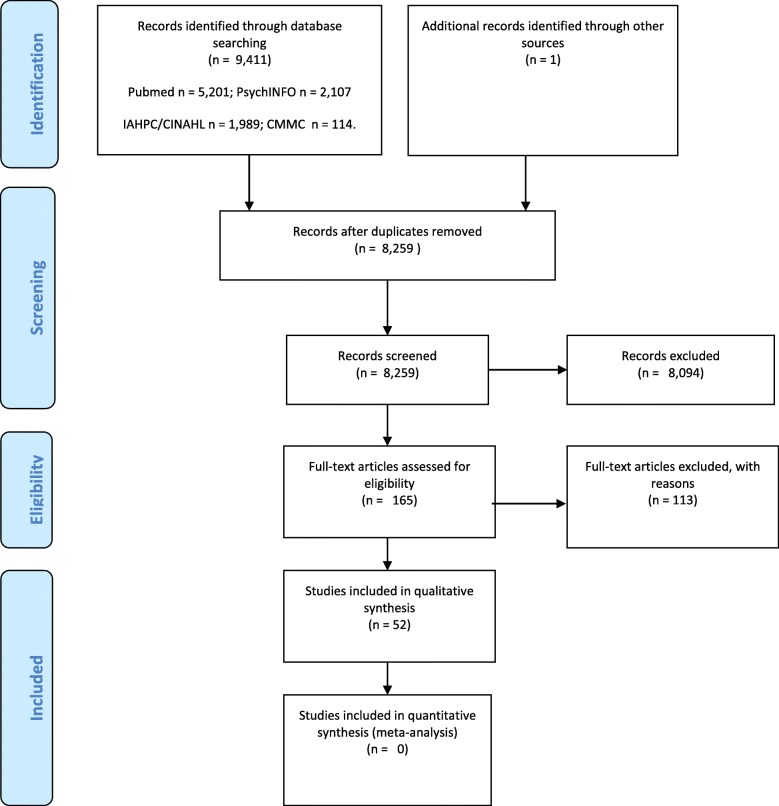
Flow diagram of study selection process (PRISMA 2009)

The search and screening process are presented in a standard diagram flow (Fig. 1). After deduplication, 8259 studies were screened to retain only original research about barriers and facilitators to the use of palliative care. Following this process, 165 studies were identified as eligible for the next selection step. Many of these studies, rather than focusing on perceived barriers or facilitators to palliative care use, had a focus on intervention evaluation or on end-of-life care in general, or on a specific area of palliative care. Fifty-two studies were retained for analysis.

### Characteristics of included studies

**Fig. 2:**
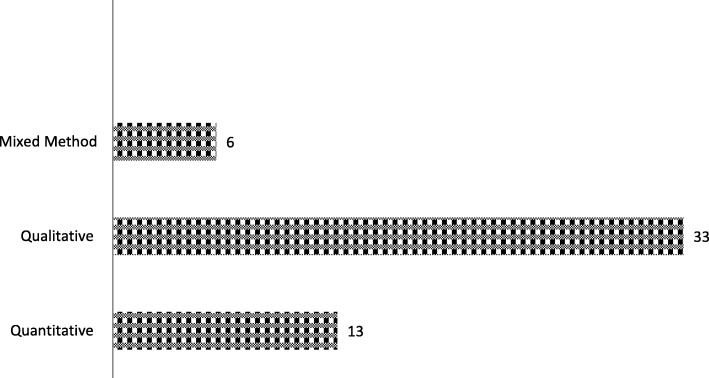
Research methods applied in the selected studies

Characteristics of included studies are displayed in Table 2. Studies were published between 1995 and 2018. Most of the studies were conducted in North America (22/52, 42.3%) or Oceania (16/52, 30.7%). In Europe 6 studies (11.6%) were conducted, as well as in Asia (6/52, 11.6%), and 1 study (1.9%) was completed in South America. Only one study (1.9%) included an international sample, in which all the continents were represented. Five studies focused uniquely on hemato-oncology (5/52, 9.6%), and the rest focused either on oncology in general or on solid tumor oncology. The studies’ populations ranged from HPs from various medical disciplines and HPs, and patients, to patients’ families. Sample sizes ranged from 1 to 984 participants. All selected studies were non-experimental, with the majority being qualitative (33/52, 63.5%), and the remaining being quantitative (cross-sectional, 13/52, 25%) or mixed-method (6/52, 11.5%; see Fig. 2).

**Table 2:** Characteristics of the selected studies

*Study no.	Author/s and year	Aim	Design	Participants and origin	Data collection method
1	Akiyama, M., Takebayashi, T., et al., 2012 [20]	To assess patients’ knowledge, beliefs, or concerns about opioids, palliative care (PC), and homecare	Quantitative – Survey study	925 outpatients with metastatic or recurrent cancer - Japan	Questionnaire (mail paper form)
2	Alaeddini, J., Julliard, K., et al., 2000 [21]	To explore physicians’ attitudes and opinions about PC and its implementation	Qualitative	23 physicians (community primary care physicians, hospital-based ambulatory clinic physicians, and specialists) - USA	Focus-group
3	Ansari, M., Rassouli, M.,et al., 2018 [22]	To explore the educational needs of stakeholders of palliative care for cancer patients	Qualitative	20 participants: cancer patients and their caregivers; healthcare providers, experts and policy-makers active in the field of cancer - Iran	Semi-structured interview
4	Beernaert, K., Deliens, L., et al., 2014 [23]	To examine barriers and facilitators of the early identification of PC needs by family physicians (FP)	Qualitative	20 FP, 12 community and PC nurses, 18 patients - Belgium	Focus-group and semi-structured interview
5	Boyd, D., Merkh, K., et al., 2011 [24]	To identify oncology nurses’ attitudes toward care at the end of life and PC use	Quantitative – Cross-sectional, descriptive correlational survey study	31 oncology nurses - USA	Questionnaire
6	Bradley, E. H., Cramer, L. D., et al., 2002 [89]	To identify physicians’ characteristics associated to referral to PC	Quantitative - Cross-sectional study	231 physicians (internists, family physicians, oncologists, pulmonologists, and cardiologists) - USA	Questionnaire
7	Broom, A., Kirby, E. et al., 2012 [25]	To examine the logics underpinning the timing of referral to PC	Qualitative	20 medical specialists (oncology, urology, haematology, geriatrics, general medicine, nonspecialist palliative medicine) - Australia	Semi-structured interview
8	Canzona, M. R., Love, D., et al., 2018 [26]	To investigate challenges that nurses face when they provide care for oncological patients transitioning from curative to palliative care and to identify educational opportunities for nurses	Qualitative Mixed	28 nurses (14 practicing in oncology and 14 practicing in palliative care) - USA	Semi-structured interview (telephone)
9	Cherny, N. I. and Catane, R., 2003 [27]	To identify oncologist-related barriers to the provision of optimal supportive and PC	Quantitative – Cross-sectional survey study	895 oncologists (members of the European Society of Medical Oncology) – Europe (82.5%), America (12.1%), Australia (2.2%), Asia (2.6%) and Africa (0.7%).	Questionnaire
10	Feeg, V. D. and Elebiary, H., 2005 [28]	To explore professionals’ perceptions about barriers related to hospice and PC, opinions about barriers related to dying at home, and barriers related to advance directives	Quantitative – Cross-sectional survey	100 national conference on PC attendees (nurses 71%, social workers 11%, hospital/hospice administrators 6%, physicians 4%, counselors 3%, chaplains 3%, and physical therapists 2%) - USA	Questionnaire
11	Fox, J., Windsor, C. et al., 2016 [29]	To explore the transition to PC	Qualitative	29 participants: patients, family carers, and healthcare professionals - Australia	Semi-structured interview
12	Gidwani, R., Nevedal, A., et al., 2017 [30]	To characterize oncologists’ perceptions of primary and specialist PC; experiences interacting with PC specialists; and the optimal interface of PC and oncology in providing PC	Qualitative	31 oncologists -USA	Semi-structured interview (telephone)
13	Gott, M., Ingleton, C. et al., 2011 [31]	To explore how transitions to a PC approach are perceived to be managed in acute hospital settings	Qualitative	58 health professionals (involved in the provision of PC) – United Kingdom	Focus group and interview
14	Groot, M. M., Vernooij-Dassen, M. J. et al., 2005 [32]	To investigate general practitioners’ task perception and barriers involved in PC	Qualitative	12–33 general practitioners (non specified the exact number) – The Netherlands	Focus-group
15	Miyashita, M., Hirai, K. et al., 2008 [33]	To investigate the barriers to referral to inpatient PC units	Qualitative	63 participants (13 advanced cancer patients, 10 family members, 20 physicians, and 20 nurses in PC and acute care cancer settings) - Japan	Semi-structured interview
16	Horlait, M., Chambaere, K. et al., 2016 [34]	To identify the barriers that oncologists experience to introduce PC to patients	Qualitative	15 oncologists Belgium	Semi-structured interview
17	Hui, D., Cerana, M. A. et al., 2016 [35]	To examine the association between oncologists’ end of life care attitudes and timely specialist PC referral	Quantitative – Cross-sectional	240 oncology specialists (120 hematologic and 120 solid oncology specialists) – USA	Questionnaire
18	Hui, D., Park, M. et al., 2015 [37]	To examine the differences in attitudes and beliefs toward PC referral between hematologic and solid tumor specialists	Quantitative - Cross-sectional	240 oncology specialists (120 hematologic and 120 solid oncology specialists) – USA	Questionnaire
19	Johnson, C., Paul, C. et al., 2011 [74]	To explore doctors’ perceptions of barriers to referring patients for specialized PC.	Qualitative – Exploratory study	40 medical doctors (general practitioners, oncologists, radiation oncologists, hematologists, respiratory physicians and colorectal surgeons) - Australia	Interview (telephone)
20	Kafadar, D., Ince, N. et al., 2015 [38]	To evaluate the managerial perspectives and opinions about specialized PC	Mixed method	70 medical directors - Turkey	Questionnaire
21	Kawaguchi, S., Mirza, R. et al., 2017 [39]	To explore medical doctors’ understanding of and experiences with PC	Qualitative	10 internal medicine residents - Canada	Semi-structured interview
22	Keim-Malpass, J., Mitchell, E. M. et al., 2015 [40]	To identify existing barriers in accessing PC services for cancer patients	Qualitative	42 clinicians, administrative support staff, and service support personnel - USA	Semi-structured interview
23	Kirby, E., Broom, A. et al., 2014 [41]	To examine how medical specialist conduct the process of negotiation of the transition to specialist PC with families	Qualitative	20 medical specialists (e.g. medical oncology, haematology, surgery, radiation oncology, general medicine, geriatrics, etc.) Australia	Semi-structured interview
24	Kumar, P., Casarett, D. et al., 2012 [42]	To identify barriers to supportive and PC services among oncology outpatients	Quantitative - Cross-sectional	313 patients with breast, lung or gastrointestinal cancer - USA	Questionnaire
25	Le, B. H., Mileshkin, C., L. et al., 2014 [77]	To explore lung cancer clinicians’ perceptions of PC and to identify views, barriers and benefits of referring to PC	Qualitative	28 clinicians (involved in the management of patients with lung cancer) - Australia	Focus group and semi-structured interview
26	Le, B. H. C. and Watt, J. N., 2010d [43]	To assess care provided to patients dying and to understand senior clinician decision-making around referral to PC	Mixed method	27 (senior) clinicians - Australia	A retrospective chart-audit and semi structured interview
27	LeBlanc, T. W., O’Donnell, J. D. et al., 2015 [44]	To examine perceptions of PC among hematologic and solid tumor oncologists	Mixed method	66 oncologists: 23 treating hematologic malignancies and 43 treating solid tumors - USA	Semi-structured interview and questionnaire
28	Llamas, K. J., Llamas, M. et al., 2001 [45]	To identify PC service needs, and educational and support needs of hospital teaching staff	Quantitative - Cross-sectional	267 multi-disciplinary oncology staff (medical, nursing, radiation therapy and other disciplines) - Australia	Questionnaire
29	Mahon, M. M. and McAuley, W. J., 2010 [46]	To examine nurses’ points of views and beliefs about PC and PC decision making	Qualitative	12 oncology nursing - USA	Interview
30	McDarby, M. and Carpenter, B. D., 2019 [64]	To identify factors that impede or facilitate the palliative care consultation team’s successful collaboration with other health care professionals	Qualitative	48 providers (19 palliative care providers, 29 nonpalliative care providers) - USA	Interview (telephone and site)
31	McGrath, P., 2013 [47]	To explore issues associated with the experience of survivorship for hematology patients	Qualitative	50 oncology patients (Multiple Myeloma, Lymphoma, Leukemia and Other) - Australia	Open-ended interview and focus group
32	McIlfatrick, S., 2007 [48]	To assess the PC needs from the perspectives of patients, informal carers and healthcare providers	Mixed method	76 patients and lay carers receiving PC services – United Kingdom	Semi-structured interview and focus-group
33	Melvin, C. S., 2010 [49]	To examine obstacles to timely referral to PC services and to explore the impact of late referral on quality of life	Qualitative	13 patients 6 family members - Australia	Interview
34	Mohammed, S., Swami, N., 2018 [50]	To examine bereaved caregivers’ experiences of providing care at home for patients with advanced cancer, while interacting with home care services	Qualitative	61 bereaved caregivers (30 intervention, 31 control) - Canada	Semi-structured interview
35	Monterosso, L., Ross-Adjie, G. M. et al., 2016 [51]	To identify HPs’ perspectives, education, and support needs related to PC provision	Mixed method	302 multi-disciplinary health professionals - Australia	Focus group
36	Norton, S. A., Wittink, M. N., et al., 2019 [72]	To explore family caregivers’ points of view of the final month of life of patients with advanced cancer	Qualitative	92 family caregivers of patients with end-stage cancer - USA	Semi-structured interview
37	O’Connor, M. and Lee-Steere, R., 2006 [52]	To explore general practitioners’ attitudes to PC in a rural center, in particular the perceived barriers to the provision of PC	Qualitative	10 general practitioners - Australia	Interview
38	Odejide, D. Y. Salas Coronado, et al., 2014 [53]	To explore hematologic oncologists’ perspectives and decision-making processes regarding end-of-life care	Qualitative	20 hematologic oncologists - USA	Focus group
39	Patel, M. I., Periyakoil, V. S., 2018 [54]	To examine clinical providers’ experiences delivering cancer care for patients at the end of life and their thoughts on potential solutions to improve quality of care	Qualitative	75 cancer care providers (35 physicians, 20 nursing staff, 12 social workers, and 8 patient navigators) - USA	Semi-structured interview
40	Philip, J. A. M. and Komesaroff, P., 2006 [55]	To explore the concept of ideal PC and the barriers to the access	Qualitative	45 PC professionals from community, inpatient, and hospital consultancy services - Australia	Focus group
41	Redman, S., White, K. et al., 1995 [90]	To examine PC nurses’ professional need and clinical knowledge	Quantitative - Cross-sectional	108 nurses - Australia	Questionnaire and interview
42	Rhee, J. J.-O., Zwar, N. et al., 2008 [56]	To establish the level of participation of urban general practitioners and to identify the barriers which they have to face in palliative care provision	Quantitative - Cross-sectional	269 general practitioners - Australia	Questionnaire
43	Rhondali, W., Burt, S. et al., 2013 [57]	To explore the oncologists’ perceptions of a supportive care program, and to determine whether renaming ‘palliative care’ influenced communication regarding referrals	Qualitative	17 oncologists - USA	Semi-structured interview
44	Rodriguez, K. L., Barnato, A. E. et al., 2007 [58]	To explore the perceptions of PC and to identify barriers to earlier use of PC in the illness trajectory	Qualitative	120 health care providers (on intensive care unit) - USA	Semi-structured interview
45	Ronaldson, S. and Devery, K., 2001 [59]	To investigate the transition to palliative care services	Qualitative	11 inpatients and 5 nursing staff members - Australia	Semi-structured interview
46	Rugno, C. P., Rebeiro Paiva, B. S. et al., 2014 [60]	To explore women’s understanding on the reasons anticancer treatment withdrawal, their thoughts about palliative care, and also prospective on the communication of bad news	Qualitative	22 women with advanced cancer (14 breast, 4 cervical, 1 ovarian, and 1 endometrial cancer) - Brazil	Semi-structured interview
47	Sanjo, M., Morita, T., 2018 [61]	To explore experiences of family members of patients with cancer receiving information concerning palliative care unit consultations	Quantitative - Survey	465 family member of adult patients with cancer - Japan	Questionnaire (mail paper form)
48	Schenker, Y., Crowley-Matoka, M. et al., 2014 [62]	To examine oncologist factors that influence referrals to outpatient specialized PC	Qualitative	74 medical oncologists - USA	Interview
49	Smith, C. B., Nelson, J. E. et al., 2012 [79]	To ascertain factors influencing physicians decisions for referral to PC	Quantitative - Cross-sectional	155 physicians (caring for cancer patients) - USA	Self-administered questionnaire
50	Walshe, C., Chew-Graham, C. et al., 2008 [91]	To examine the influences on referral decisions made (within community PC services)	Qualitative	57 healthcare professionals interviewed; 13 case notes; 84 other non-patient documents – United Kingdom	Interview, observation and documentary analysis
51	Ward, A. M., Agar, M. et al., 2009 [63]	To explore attitudes of medical oncologists toward collaboration with specialist PC services	Mixed method	78 medical oncologists and 37 trainees – Australia	Questionnaire (web-based)
52	Zhang, Z. and Cheng, W.W., 2014 [65]	To explore the process to access and role of PC	Qualitative	1 patient (doctor) – China	Observation

### Thematic analysis

**Fig. 3:**
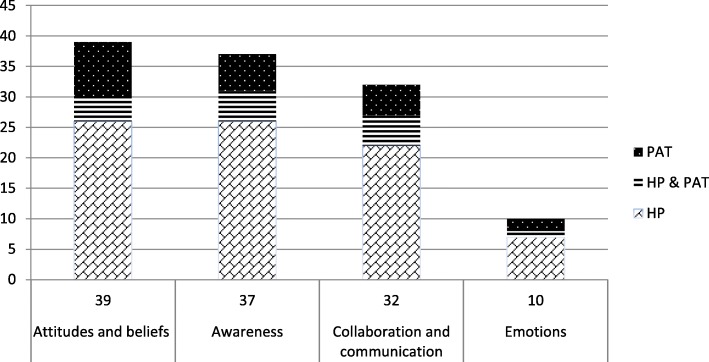
Themes emerged according to the participant population (PAT: patients and/or their family; HP: health care professionals)

Four themes emerged: awareness of palliative care, collaboration and communication in palliative care-related settings (e.g., hospital oncological wards), attitudes and beliefs towards palliative care, and emotions involved in disease pathways among HPs, patients and their families (see Table 1, Fig. 3, and Table 3 in the Additional file 2). The following categorization was developed in order to avoid potential overlapping barriers or facilitators between different themes. In particular, we included in the theme “awareness”, barriers and facilitator related to knowledge and understanding (e.g. resulting from the participants’ assessment of knowledge about palliative care), and those related to education (resulting from courses, practical trainings or experience in palliative care units or related settings). We included in the theme “attitudes and beliefs”, all those barriers and facilitators reported as attitudes or beliefs in the relevant studies, and, additionally, all other aspects related to opinions, views, thoughts, rules, assumptions, interpretations, ideas (regarded as absolute truths) [66] and subjective evaluations (that range from good to bad) that are represented in memory [67]. In the theme “Emotions”, we included barriers or facilitators referring to emotions, in particular referring to primary emotions (e.g. anger, sadness, fear, and surprise), secondary emotions (e.g. optimism), and tertiary emotions (e.g. frustration) [68, 69]. In the theme “Communication and collaboration” barriers and facilitators referring to collaboration as any mutually beneficial and well-defined relationship entered into by two or more people to achieve common goals [70] and those referring to transferring information, sharing meaning, and boundary negotiation [71]. Often a single study identified numerous barriers or facilitators; therefore, the number of barriers and facilitators is greater than the total number of the studies included in each theme. In the following section the results are presented by actor involved in palliative care use pathways: barriers and facilitators related to healthcare professional, those related to patient and their family and, finally, those related to the relationship between HPs and patients and family.

### Barriers and facilitators related to health care professionals

This section reports barriers and facilitators related to HPs. Most studies included only HPs (37/52, 71.2%) and the rest included HPs and patients and family (15/52, 28.8%). HPs included were mostly medical doctors, including residents, internists, GPs, palliative care doctors, oncologists, hemato-oncologists (and other specialties), and nurses. Only a few studies included other health-related professions, such as social workers or counselors.

#### Awareness of palliative care

Within this theme we included studies on knowledge of palliative care (e.g., knowledge of palliative care’s scope and benefits), education on palliative care, and experience in the palliative care field (e.g., palliative care training or practical experience). In most of these studies (e.g., [22, 39, 46]), HPs’ lack of knowledge of palliative care in general (e.g. how and when initiate palliative care, and lack of familiarity with the basic principles of palliative care, including pain management, among medical students), or lack of one or more areas pertaining to it, such as existential issues [23] represent a serious obstacle to palliative care utilization. Some studies also reported a lack of understanding of palliative care services [22, 25, 43, 54], their scope [29, 64], or their applicability [51, 64]. Others showed a lack of knowledge of locally available palliative care services among HPs [22, 62]. Often physicians (e.g., oncologists), nurses, and other HPs specifically reported a lack of education in palliative care [21, 22, 40, 45, 51, 64]. Other studies reported a lack of practical training [22, 27, 34, 64] or working experience [23, 34] in palliative care.

Potential facilitators to palliative care use were identified; some studies showed that the provision of education on palliative care, including education on the initiation phase, improved utilization [38, 39, 46, 52, 58, 64]. One study focused on family physicians and highlighted that the use of specific tools for palliative care needs assessment was useful to address those needs in a timely manner [23]. Finally, several of quantitative studies showed that previous experience, general knowledge of end-of-life care, palliative care education provision for HPs (e.g., workshops and practical training) [35, 51, 52, 58, 64] as well as clinical guidelines on palliative care delivery [51] led to greater utilization of palliative care services.

#### Collaboration and communication between health care professionals

Eighteen studies reported poor communication between HPs, particularly between palliative care specialists and oncologists [30]. Reasons to explain this suboptimal communication included poor information exchange [22, 48, 72], lack of effective team communication (e.g., concerning communication of the prognosis between palliative care specialists and oncologists) [45, 64], as well as lack of clarity [63] and consensus [34, 48, 73] on palliative care initiation. Ineffective communication can be due to lack of opportunity of conversations between cancer care and palliative care providers [64]. A study showed that the relationships between cancer and palliative care providers solely focused on patient care not representing trusted relationships which facilitate collaboration [64].

Several potential facilitators, such as improving collaboration between oncologists and the palliative care team [30], involving other HPs (e.g., nurses) in the negotiation phase with patients and their family, and joining other HPs at multidisciplinary meetings [23], were reported to improve palliative care use. A study reported that palliative care team strategic visibility in patient floors and hospital-wide events, as well as unintentional visibility, such as being present around the hospital, would nurture casual relationships between palliative care team and nonpalliative providers and hinder palliative care consultations [64]. Additionally, this study highlighted that an active marketing of the utility of palliative care consultations and education about the expertise of palliative care team to nonpalliative providers, as well as providing scientific support for recommendations during consultations represent facilitators for the receipt new palliative care consultations [64].

#### Emotions involved in cancer care pathways

Six qualitative studies reported that HPs in oncological and hemato-oncological settings had issues with managing the emotions involved in the discussions of diagnosis, prognosis and palliative care options with patients and families. In the selected studies, emotional discomfort with death and dying [32, 53], discomfort preceded by a feeling of failure and defeat (when palliative care discussion is brought up or when referring to palliative care) [34, 63], difficulty in managing the close emotional bond of patients or family members (sometimes resulting in personal identification with the patient) [26, 34, 52], and difficulty in addressing their reactions to prognosis disclosure (e.g. patient shock or anger) [26, 52] were reported to be experienced by HPs. From these studies a lack of available psychological support for HPs who experience an high emotional involvement emerged [34, 52]. Narrative-based training for HPs, in particular for nurses, may represent a facilitator to properly process emotions during discussions of diagnosis, prognosis and palliative care options, additionally, to help building therapeutic alliances with patients and colleagues, and to improve communication skills [26].

#### Attitudes and beliefs towards palliative care

Thirty-four studies identified attitudes and beliefs towards palliative care, which may represent barriers or facilitators to palliative care utilization. Common beliefs were the association of palliative care or hospice with death [28, 51], the inappropriateness of palliative care for complex problems if physical symptoms were not present [38, 74], the only palliative care goal being symptom management [46, 74], incompatibility of palliative care and cancer therapy [57, 62], suitability of palliative care for certain types of cancer such as lung cancer (less suitable for others e.g., breast cancer) [42], and suitability for older patients [25, 53]. In addition, specific beliefs among hemato-oncologists included inappropriateness of palliative care for onco-hematologic patients (e.g., specific needs, such as transfusions, not being addressed) [44, 53], the development of palliative care services specifically for solid tumor cancer rather than for blood cancer [53] and talking about palliative care at an early stage not being appropriate due to the large amount of other issues to address [47]. Additionally, some studies found that hemato-oncologists perceived palliative care as end-of-life care, preferred to maintain control also on palliative care, and had lack of trust in other professionals’ oncology knowledge [44]. Moreover, further studies showed that hemato-oncologists had lower comfort levels towards palliative care referral compared to oncologists [35] and were conducive to referral with the service name supportive care instead of palliative care [36]. In these studies the difficulty of accepting death and avoiding discussing death was reported [25, 29, 34]. A recent study showed how HPs felt compelled to fully exhaust disease-directed treatment options prior to referring patients to palliative care providers [54]. HPs’ beliefs in the selected studies also included earlier palliative care introduction in the disease trajectory representing a source of confusion over roles [75], increasing care fragmentation [75], and threat to palliative care patient autonomy [45, 58]. Overall, attitudes of HPs included beliefs about losing patient care management [25, 44, 52], unconsciously avoiding admitting failure [25, 63, 64], personal crusades for the disease [76], cure-focused attitudes and over-emphasis on the treatment [34, 44, 45, 54], palliative care incompatibility with the hospital life-saving culture, including beliefs that hospital reputation can be damaged [58], and considering palliative care referring as the last resort [64]. Other studies found that oncologists were reluctant to refer to the palliative care team [28, 37], due to a negative view of the palliative care team involvement [27, 30, 44, 45, 77] or past negative experiences [44, 77]. A qualitative study highlighted that nonpallitive providers were reluctant to refer to palliative care team for consultations because they felt capable of providing palliative care, without the involvement of the palliative care team, or because they were hesitant about specific medications for pain/symptom management prescribed by the palliative care team [64]. Other reasons for the lack of palliative care referral included beliefs that patients or their family might have inappropriate reactions to the referral [54, 77], that discussions or referral upset patients and families [34, 54, 75], or simply because of a lack of interest in palliative care [38, 56]. Attitudes which might represent facilitators to palliative care use, in particular palliative care provision and palliative care needs identification, were the hospital team paying attention to ethical differences and acting according to patient values [21, 23].

### Barriers and facilitators related to the relationship between health care professionals and patients

This section shows barriers and facilitators for both HPs and patients and families, with a focus on communication issues between HPs and patients and/or families. Seventeen studies reported on these issues.

#### Collaboration and communication between health care professionals and patients and/or their families

A large number of the selected studies’ samples included doctors (20/52 [38.5%], including oncologists, hemato-oncologists, GPs, and residents), followed by HPs (17/52 [32.7%] that included various health professions including nurses), and patients and families (15/52 [28.8%], including studies that also included HPs). Some studies reported difficulties in negotiating the transition to palliative care [29] which was chaotic and frequently occurred a short time prior to death [72], due to lack of open communication between HPs and patients and families [31, 55, 72, 74], as well as lack of information on patient prognosis [22, 72] or available support and services [61, 72, 74]. A study found that long-term relationships between hemato-oncologists and patients result in considerable difficulty conducting end-of-life discussions [53]. Three recent studies showed that further barriers to palliative care use included receiving conflicting information on goals of care by HPs [26, 61, 72], lack of shared perspectives on patient conditions by HPs [72], no discussions on end of life between patients and family and oncologists [54, 72], no preparation of the patient and family to the imminent deterioration of the patient conditions [72]. Several aspects related to the lack of open communication were reported in these studies, such as absence of training in communication skills [23, 34], problems with communication of a bad prognosis [45, 54, 78], lack of communication skills [22, 61] and difficulty discussing spiritual, psychosocial, and emotional issues [52]. Lack of effective communication and collaboration between HPs and patients and families [31, 39, 55] and not using communication tools [23] were further obstacles to palliative care utilization. From some studies it emerged that collaboration and communication could be influenced by the unwillingness of the patient’s family to openly communicate with the patient about diagnosis and prognosis (thus influencing the communication between the HPs and the patient) [55] as well as language or cultural barriers [34] or family emotional reactions [34], and difficulty in dealing with these for HPs [26]. Finally, a patient with impaired communication ability might represent a barrier to early identification of palliative care needs [23].

On the contrary, early identification of palliative care needs could be facilitated by effective communication [43], a gentle exploration of the topic, and the use of communication notebooks and medical files with non-physical information by the GP [23], as well as ‘reinforcing’ the relationship with the patient (e.g., making regular contacts with him/her or creating trust in the patient) [23, 64]. Additionally, designing proactive approaches for non-face-to-face communication with patients and family [54], interacting and collaborating with home care professionals by patients and family (home palliative care services) [50] and conducting regular follow-ups, with direct contact with patients, by the palliative care team [64].

Moreover, providing information on the medical condition and palliative care options to the patients or family [51, 65], the family involvement in care trajectories and decision-making [65, 76], medical language translated into more simple language for patients and families [76], and HPs receiving training on communication skills [23, 26], were reported as aspects that improved palliative care utilization (provision or increased palliative care referrals). Additionally, training in communication skills could improve transition to specialized palliative care [41]. Finally, some studies showed that proposing palliative care with a new name (‘supportive care’) might increase palliative care services referral [37, 57].

### Barriers and facilitators related to patients and their families

Only a limited number of studies included patients and/or families in the sample (only patients and/or families, 10/52 [19.2%]; patients and/or families and HPs, 5/52 [9.6%]). However, some studies that included only HP samples also reported perceived barriers and facilitators to palliative care use among patients and their families and these are also reported below.

#### Awareness of palliative care

Eleven studies which showed lack of palliative care knowledge in the patient population and in families were identified. In particular, low awareness about palliative care [38, 42, 51], and lack of knowledge of palliative care and local services [33, 46, 61, 64] were recurrent barriers. Other studies reported uncertainty about palliative care scope [30], difficulty in defining palliative care [48], and lack of patient education [40] or lack of guidelines for patient education [22] as obstacles to palliative care use. Recent studies reported lack of information on hospital and home palliative care services by HPs [61] and difficulty in navigating the home palliative care system among patients [50] as barriers to palliative care use.

A greater level of education in patients, including increased knowledge of palliative care benefits [38, 47, 64] and demonstration of palliative care utility via exposure [64], provision of clear information on end of life [50], with the integration of a lay health coach into oncology teams [54] has been found to be associated with greater palliative care use.

#### Emotions involved in cancer care pathways

Some qualitative studies have shown that emotions involved (e.g., sadness and suppression) and psychological reactions (e.g., unrealistic optimism and unrealistic expectations especially among hemato-oncology patients [53]) in the disease path experienced by the patients and families might make early identification of palliative care needs difficult [23] as well as palliative care referrals [34]. Recent studies highlighted that feelings of frustration and anger due to unpreparedness of the family to patient transition to palliative care [72], fear of palliative care [59], and resistance or denial to accept the actual medical condition and/or palliative care options [25, 64] represent barriers. In particular, one case study highlighted how assessing the psychological condition, in particular patients’ emotions, contributed to improved palliative care utilization [65].

#### Attitudes and beliefs towards palliative care

This theme emerged in eighteen studies. The most recurrent themes were family or patient reluctance [34, 63], willingness to continue the active treatment [72], a negative view of palliative care [33, 34, 75], misconceptions about palliative care [39, 51], palliative care being viewed as terminal care [20, 49, 59], and beliefs about losing hope [24, 34, 77] or differing perspectives about hope (overoptimism versus hopelessness) when discontinuation of active treatment was communicated [60]. A qualitative study reported that patients wished to receive care from family physicians or nurses rather than from unknown HPs in a palliative care unit [33]. Additional beliefs reported by the selected studies were the perception of palliative care as a lesser care alternative and cost-saving option [58], and palliative care options which must not be brought up at the diagnosis or prognosis discussions [47, 79]. Patient or family attitudes that were reported included infinite trust and faith in medicine [34], as well as family forbidding discussion of palliative care during conversations about diagnosis or prognosis [55]. On the contrary, one study found that patients’ openness to their own needs was reported as a facilitator to early identification of palliative care needs [23].

## Discussion

This study systematically reviewed current literature on using palliative care in oncology and hemato-oncology. The focus was on cognitive barriers and facilitators influencing the use of palliative care services from the perspectives of HPs, adult patients with cancer, and their families. Literature on this topic is very heterogeneous in objectives and focus, mainly addressing palliative care use, palliative care provision, and palliative care service referral. Findings show that barriers and facilitators can be summarized in four areas: *awareness of palliative care*; *collaboration and communication in palliative care-related settings*; *attitudes and beliefs towards palliative care;* and *emotions involved in disease pathways*. To note that no relevant differences regarding the themes emerging in the most recent studies, conducted between 2009 and 2019, and in the previous studies, conducted between 1995 and 2008, were observed. Overall, the studies revealed that lacking awareness of palliative care, having incorrect beliefs, negative attitudes and negative emotions towards palliative care among HPs or patients and families, as well as lack of collaboration and communication skills among HPs, poor communication and consensus on palliative care between HPs and palliative care team, and lack of open communication between HPs and patients and families represented the most common barriers to providing, asking for PC consultations or referring to, and consequently using palliative care services. In addition, it should be noted that specific barriers in hemato-oncology include HPs’ lack of trust regarding the appropriateness of palliative care services for hemato-oncology patients, and lower comfort levels towards palliative care referrals compared to oncologists. These studies suggested that enhancing HPs’ education about and clinical experience of palliative care as well as patients and families’ education could facilitate palliative care utilization.

This study deeply exploring cognitive barriers and facilitators to palliative care provided an adequate understanding and specific nuances regarding palliative care access. In particular, this study showed that emotions play a key role for palliative care utilization, not only among patients and their families, but also among healthcare professionals. Moreover, this work highlighted that not only collaboration and communication between HPs contribute significantly to palliative care utilization, but also collaboration and communication between HPs and patients, and their families. With this regards, we strongly believe that the information on cognitive barriers and facilitators is highly relevant due to the fact that these represent directly addressable aspects. By intervening on cognitive barriers and facilitators potentially leads to directly enhance the utilization of palliative care services, as well as to partially overcome the other two types of barriers (e.g. more general awareness of palliative care benefits, including awareness among local health authority services, can encourage changes at systematic and organizational levels towards palliative care services optimization).

A way to interpret the meaning and the implications of these results refers back to the idea that health professionals, and medical doctors especially, are trained in the so-called curative model. In the curative model, health care practitioners treat patients with the sole intent of curing them, and do not consider other aspects such as reducing pain or stress [80]. By definition, palliative care has no curative goal, and intends neither to hasten nor postpone death [13]. What it can and should achieve is to improve the quality of life [13]. Our review suggests that cure-focused cultural aspects in health care settings may constitute a barrier to palliative care provision and referral. In particular, the predominance of a curative model [23, 34] may explain the incorrect belief about palliative care as mostly conceived only to address physical symptoms [46]. In other cases, the cure-focused culture may influence the HPs’ perception and confidence towards palliative care and its team’s holistic approach [81]. Confidence was reported as being low or lacking among HPs [77], in line with a recent study [82], mainly because palliative care was seen as ‘external’ to the cure-focused culture. In agreement with this, a recent American study showed that the palliative care team was perceived as being a ‘team of outsiders’ by oncologists [30]. In this cure-focused culture, dealing with emotions [32, 53], being highly involved in chronic conditions as well as discussing spiritual issues [52] may be very challenging. Therefore, a discrepant view about palliative care utility [43], no consensus on appropriate timing of palliative care service initiation [48, 74] as well as poor information exchange [30, 48] have all been found to make the negotiation of palliative care transition extremely arduous [29].

### Limitations

Although this is one of the few systematic reviews that included studies at any time and from any country with a specific focus on cognitive barriers and facilitators to palliative care, limitations exist.

First, we included non-interventional studies, whose quality should be considered lower than that of interventional [83]. However, due to our explorative aim, they were the most appropriate to answer our research question. Second, integrating qualitative and quantitative studies in one review was a challenge. However, both qualitative and quantitative results increased our understanding of the factors involved in palliative care utilization, the qualitative results gave a context for these factors from the perspectives of patients, formal and informal caregivers. This is a relatively new path of research which we have enriched with the present study. Finally, the inclusion of studies focused on palliative care in general, rather than also including studies on the specific palliative care areas (e.g., spiritual care) may represent a limitation. However, the scope of this review was to focus on palliative care as a typology of services, and explore how HPs, patients and families perceived palliative care as a whole.

### Directions for future research

Most of the studies included in the review were conducted in the USA and Oceania, leaving space for international, European, African, and Asian studies. This is necessary due to country-specific characteristics of health care systems and cultures (e.g., beliefs, religion) which influence palliative care services’ utilization, as this review showed.

Terminology issues emerged while conducting this review, due mainly to lack of consistency in the use of the term palliative care, hospice care and related terms across countries and over time. Making consistent the terms so that the meaning of palliative care is equal all around the globe is necessary for public health services to be informed by relevant studies and in order to make study comparisons possible.

Palliative care use between ‘solid’ and ‘liquid’ cancers might be quite different in terms of timing. Even though a small number of studies investigating palliative care in hemato-oncology exist, most literature concentrates on ‘solid’ oncology, leaving room for more research in this area.

Overall, there was a limited variety in participant sample types, as they were mostly composed of medical doctors or nurses, missing the perspectives of patients and families. A large number of studies on factors influencing palliative care use for patients included only HP samples, rather than patients and families. In future, qualitative studies especially need to explore these factors by making patients and families’ voices heard.

### Clinical implications

One of the main reasons impeding patients with oncological conditions experiencing physical suffering and high distress, who certainly would benefit from palliative care services, to use these can be attributed to the lack of collaboration and communication skills necessary to properly address discussions with patients and family, as well as to the general stigma attached to palliative care. Therefore, discussion of palliative care options is often avoided, especially due to lack of openness of communication on diagnosis and prognosis between patients and family [55], or between HPs and patients [31, 45]. Training to increase collaboration and communication skills in palliative care settings should be available to HPs [84, 85]. In addition, it might be relevant to implement the use of relevant tools for disclosing unfavorable information, such as SPIKES [80] or BREAKS [86], and to implement guidelines and recommendations for how doctors should prepare to delivering bad news [86, 87].

Second, studies reported that the trajectory of the illness might be an obstacle to palliative care referral. In line with the principle of co-existence between disease treatment and palliative care, triggers for referral to palliative care services should be activated when transitions in care are verified (e.g., metastases discovered) or by the emergence of distress recognized with specific screening tools [3]. Therefore, referral for specialist palliative care support should be conducted at any time when physical, psychological, social or spiritual unmet needs cannot be satisfactorily resolved by the primary care team, even when the main goal of disease management is curative in intent. Interprofessional collaboration skills (e.g. to learn to be comfortable with performance review by team members of different professional background and with dilution of the traditional one-to-one relationship with patient [88]) should be enhanced.

Overall, strategies to encourage collaboration between oncologists and the palliative care team, to define a clear division of responsibility, and to encourage sharing of expertise of nonphysician palliative care team members should be applied in order to improve the interface between oncologists and palliative care providers [30].

## Conclusions

Being aware of the existing cognitive barriers to palliative care utilization can help to address them in practice, particularly when strategies aimed at increasing the recognition of the importance of palliative care and at introducing it early on to patients are implemented. This would provide patients with continuity of care, allowing them to gain the possible benefits from palliative care when they are in need.

## Supplementary information

**Additional file 1:.**

**Additional file 2:.**

## Data Availability

The datasets used and/or analyzed during the current review are available from the corresponding author on reasonable request.

## Abbreviations

PC: Palliative care
HPs: Healthcare professionals
FP: Family physician

## References

[1] WHO (2014). Strengthening of palliative care as a component of comprehensive care throughout the life course - World Health Assembly. In.

[2] Dumanovsky T, Augustin R, Rogers M, Lettang K, Meier DE, Morrison RS (2016). The growth of palliative care in US hospitals: a status report. J Palliat Med.

[3] Hawley P (2017). Barriers to access to palliative care. Palliat Care.

[4] Carrillo JE, Carrillo VA, Perez HR, Salas-Lopez D, Natale-Pereira A, Byron AT (2011). Defining and targeting health care access barriers. J Health Care Poor Underserved.

[5] Slort W (2014). General practitioner-patient communication in palliative care.

[6] Donkor A, Luckett T, Aranda S, Phillips J. Barriers and facilitators to implementation of cancer treatment and palliative care strategies in low-and middle-income countries: systematic review. Int J Public Health. 2018;63(9):1047–57. 10.1007/s00038-018-1142-2 29974131

[7] Ahmed N, Bestall J, Ahmedzai SH, Payne S, Clark D, Noble B (2004). Systematic review of the problems and issues of accessing specialist palliative care by patients, carers and health and social care professionals. Palliat Med.

[8] Carmont S-A, Mitchell G, Senior H, Foster M. Systematic review of the effectiveness, barriers and facilitators to general practitioner engagement with specialist secondary services in integrated palliative care. BMJ Support Palliat Care. 2018;8(4):385–99. 10.1136/bmjspcare-2016-001125 28196828

[9] Love AW, Liversage LM (2014). Barriers to accessing palliative care: a review of the literature. Prog Palliat Care.

[10] CfRaD CRD (2008). Systematic reviews - CRD’s guidance for undertaking reviews in health care: University of York.

[11] Moher D, Liberati A, Tetzlaff J, Altman DG (2010). Preferred reporting items for systematic reviews and meta-analyses: the PRISMA statement. Int J Surg.

[12] Lucas PJ, Baird J, Arai L, Law C, Roberts HM (2007). Worked examples of alternative methods for the synthesis of qualitative and quantitative research in systematic reviews. BMC Med Res Methodol.

[13] Mousing CA, Timm H, Lomborg K, Kirkevold M (2018). Barriers to palliative care in people with chronic obstructive pulmonary disease in home care: a qualitative study of the perspective of professional caregivers. J Clin Nurs.

[14] **WHO Definition of Palliative Care** [http://www.who.int/cancer/palliative/definition/en/].

[15] Groh G, Feddersen B, Führer M, Borasio GD (2014). Specialized home palliative care for adults and children: differences and similarities. J Palliat Med.

[16] Peretti-Watel P, Bendiane MK, Moatti JP (2005). Attitudes toward palliative care, conceptions of euthanasia and opinions about its legalization among French physicians. Soc Sci Med.

[17] Hui D, De La Cruz M, Mori M, Parsons HA, Kwon JH, Torres-Vigil I, Kim SH, Dev R, Hutchins R, Liem C (2013). Concepts and definitions for “supportive care,”“best supportive care,”“palliative care,” and “hospice care” in the published literature, dictionaries, and textbooks. Support Care Cancer.

[18] Pastrana T, Jünger S, Ostgathe C, Elsner F, Radbruch L (2008). A matter of definition–key elements identified in a discourse analysis of definitions of palliative care. Palliat Med.

[19] Popay J, Roberts H, Sowden A, Petticrew M, Arai L, Rodgers M, Britten N, Roen K, Duffy S (2006). Guidance on the conduct of narrative synthesis in systematic reviews. A product from the ESRC methods programme Version.

[20] Akiyama M, Takebayashi T, Morita T, Miyashita M, Hirai K, Matoba M, Akizuki N, Shirahige Y, Yamagishi A, Eguchi K (2012). Knowledge, beliefs, and concerns about opioids, palliative care, and homecare of advanced cancer patients: a nationwide survey in Japan. Supportive Care Cancer.

[21] Alaeddini J, Julliard K, Shah A, Islam J, Mayor M (2000). Physician attitudes toward palliative care at a community teaching hospital. Hosp J.

[22] Ansari M, Rassouli M, Akbari ME, Abbaszadeh A, Sari AA (2018). Educational needs on palliative Care for Cancer Patients in Iran: a SWOT analysis. Int J Community Based Nurs Midwifery.

[23] Beernaert K, Deliens L, De Vleminck A, Devroey D, Pardon K, Van den Block L, Cohen J (2014). Early identification of palliative care needs by family physicians: a qualitative study of barriers and facilitators from the perspective of family physicians, community nurses, and patients. Palliat Med.

[24] Boyd D, Merkh K, Rutledge DN, Randall V (2011). Nurses' perceptions and experiences with end-of-life communication and care. Oncol Nurs Forum.

[25] Broom A, Kirby E, Good P, Wootton J, Adams J (2012). Specialists’ experiences and perspectives on the timing of referral to palliative care: a qualitative study. J Palliat Med.

[26] Canzona MR, Love D, Barrett R, Henley J, Bridges S, Koontz A, Nelson S, Daya S (2018). "operating in the dark": Nurses' attempts to help patients and families manage the transition from oncology to comfort care. J Clin Nurs.

[27] Cherny NI, Catane R (2003). Attitudes of medical oncologists toward palliative care for patients with advanced and incurable cancer: report on a survery by the European Society of Medical Oncology Taskforce on palliative and supportive care. Cancer.

[28] Feeg VD, Elebiary H (2005). Exploratory study on end-of-life issues: barriers to palliative care and advance directives. Am J Hosp Palliat Med.

[29] Fox J, Windsor C, Connell S, Yates P (2016). The positioning of palliative care in acute care: a multiperspective qualitative study in the context of metastatic melanoma. Palliat Support Care.

[30] Gidwani R, Nevedal A, Patel M, Blayney DW, Timko C, Ramchandran K, Kelly PA, Asch SM (2017). The appropriate provision of primary versus specialist palliative care to Cancer patients: Oncologists' perspectives. J Palliat Med.

[31] Gott M, Ingleton C, Bennett MI, Gardiner C (2011). Transitions to palliative care in acute hospitals in England: qualitative study. BMJ Br Med J.

[32] Groot MM, Vernooij-Dassen MJ, Crul BJ, Grol RP (2005). General practitioners (GPs) and palliative care: perceived tasks and barriers in daily practice. Palliat Med.

[33] Miyashita M, Hirai K, Morita T, Sanjo M, Uchitomi Y, Miyashita M, Hirai K, Morita T, Sanjo M, Uchitomi Y (2008). Barriers to referral to inpatient palliative care units in Japan: a qualitative survey with content analysis. Support Care Cancer.

[34] Horlait M, Chambaere K, Pardon K, Deliens L, Belle S, Van Belle S (2016). What are the barriers faced by medical oncologists in initiating discussion of palliative care? A qualitative study in Flanders, Belgium. Supportive Care Cancer.

[35] Hui D, Cerana MA, Minjeong P, Hess K, Bruera E (2016). Impact of Oncologists' attitudes toward end-of-life care on Patients' access to palliative care. Oncologist.

[36] Hui D, Bansal S, Park M, Reddy A, Cortes J, Fossella F, Bruera E (2015). Differences in attitudes and beliefs toward end-of-life care between hematologic and solid tumor oncology specialists. Ann Oncol.

[37] Hui D, Park M, Liu D, Reddy A, Dalal S, Bruera E (2015). Attitudes and beliefs toward supportive and palliative care referral among hematologic and solid tumor oncology specialists. Oncologist.

[38] Kafadar D, Ince N, Akcakaya A, Gumus M (2015). Evaluation of managerial needs for palliative care centers: perspectives of medical directors. Asian Pac J Cancer Prev.

[39] Kawaguchi S, Mirza R, Nissim R, Ridley J (2017). Internal medicine residents' beliefs, attitudes, and experiences relating to palliative care: a qualitative study. Am J Hosp Palliat Med.

[40] Keim-Malpass J, Mitchell EM, Blackhall L, DeGuzman PB (2015). Evaluating stakeholder-identified barriers in accessing palliative care at an NCI-designated cancer center with a rural catchment area. J Palliat Med.

[41] Kirby E, Broom A, Good P, Wootton J, Adams J (2014). Families and the transition to specialist palliative care. Mortality.

[42] Kumar P, Casarett D, Corcoran A, Desai K, Li Q, Chen J, Langer C, Mao JJ (2012). Utilization of supportive and palliative care services among oncology outpatients at one academic Cancer center: determinants of use and barriers to access. J Palliat Med.

[43] Le BHC, Watt JN (2010). Care of the dying in Australia’s busiest hospital: benefits of palliative care consultation and methods to enhance access. J Palliat Med.

[44] LeBlanc TW, O'Donnell JD, Crowley-Matoka M, Rabow MW, Smith CB, White DB, Tiver GA, Arnold RM, Schenker Y (2015). Perceptions of palliative care among hematologic malignancy specialists: a mixed-methods study. J Oncol Pract.

[45] Llamas KJ, Llamas M, Pickhaver AM, Piller NB (2001). Provider perspectives on palliative care needs at a major teaching hospital. Palliat Med.

[46] Mahon MM, McAuley WJ (2010). Oncology nurses' personal understandings about palliative care. Oncol Nurs Forum.

[47] McGrath P (2013). End-of-life care in hematology: update from Australia. J Soc Work End Life Palliat Care.

[48] McIlfatrick S (2007). Assessing palliative care needs: views of patients, informal carers and healthcare professionals. J Adv Nurs.

[49] Melvin CS (2010). Patients' and families' misperceptions about hospice and palliative care: listen as they speak. J Hosp Palliat Nurs.

[50] Mohammed S, Swami N, Pope A, Rodin G, Hannon B, Nissim R, Hales S, Zimmermann C (2018). 'I didn't want to be in charge and yet I was': bereaved caregivers' accounts of providing home care for family members with advanced cancer. Psycho-Oncology.

[51] Monterosso L, Ross-Adjie GM, Rogers IR, Shearer FM, Rogers JR (2016). How well do we understand health care professionals' perceptions and needs in the provision of palliative care? A mixed methods study. J Palliat Med.

[52] O'Connor M, Lee-Steere R (2006). General practitioners' attitudes to palliative care: a Western Australian rural perspective. J Palliat Med.

[53] Odejide OO, Salas Coronado DY, Watts CD, Wright AA, Abel GA (2014). End-of-life care for blood cancers: a series of focus groups with hematologic oncologists. J Oncol Pract.

[54] Patel MI, Periyakoil VS, Moore D, Nevedal A, Coker TR (2018). Delivering end-of-life Cancer care: perspectives of providers. Am J Hosp Palliat Care.

[55] Philip JAM, Komesaroff P (2006). Ideals and compromises in palliative care. J Palliat Med.

[56] Rhee JJ-O, Zwar N, Vagholkar S, Dennis S, Broadbent AM, Mitchell G (2008). Attitudes and barriers to involvement in palliative care by Australian urban general practitioners. J Palliat Med.

[57] Rhondali W, Burt S, Wittenberg-Lyles E, Bruera E, Dalal S (2013). Medical oncologists' perception of palliative care programs and the impact of name change to supportive care on communication with patients during the referral process. A qualitative study. Palliat Supportive Care.

[58] Rodriguez KL, Barnato AE, Arnold RM (2007). Perceptions and utilization of palliative Care Services in Acute Care Hospitals. J Palliat Med.

[59] Ronaldson S, Devery K (2001). The experience of transition to palliative care services: perspectives of patients and nurses. Int J Palliat Nurs.

[60] Rugno FC, Ribeiro Paiva BS, Nunes JS, Paiva CE (2014). 'There won't' be anything else … it's over': perceptions of women referred to palliative care only. Eur J Oncol Nurs.

[61] Sanjo M, Morita T, Miyashita M, Sato K, Kamibeppu K, Tsuneto S, Shima Y (2018). Are bereaved family members satisfied with information provision about palliative care units in Japan?. Am J Hosp Palliat Care.

[62] Schenker Y, Crowley-Matoka M, Dohan D, Rabow MW, Smith CB, White DB, Chu E, Tiver GA, Einhorn S, Arnold RM (2014). Oncologist factors that influence referrals to subspecialty palliative care clinics. J Oncol Pract.

[63] Ward AM, Agar M, Koczwara B (2009). Collaborating or co-existing: a survey of attitudes of medical oncologists toward specialist palliative care. Palliat Med.

[64] McDarby M, Carpenter BD (2019). Barriers and facilitators to effective inpatient palliative care consultations: a qualitative analysis of interviews with palliative care and nonpalliative care providers. Am J Hosp Palliat Med.

[65] Zhang Z, Cheng W-W (2014). Communication as an approach to resolve conflict about the implementation of palliative care. Am J Hosp Palliat Med.

[66] Dozois D, Beck A (2008). Chapter 6-cognitive schemas, beliefs and assumptions. Risk Factors in Depression San Diego: Elsevier.

[67] Harmon-Jones E, Harmon-Jones C, Amodio DM, Gable PA (2011). Attitudes toward emotions. J Pers Soc Psychol.

[68] Shaver P, Schwartz J, Kirson D, O'connor C (1987). Emotion knowledge: further exploration of a prototype approach. J Pers Soc Psychol.

[69] Parrott WG (2001). Emotions in social psychology: essential readings: psychology press.

[70] Green BN, Johnson CD (2015). Interprofessional collaboration in research, education, and clinical practice: working together for a better future. J Chiropr Educ.

[71] O'Rourke M, Crowley S, Eigenbrode SD, Wulfhorst J (2013). Enhancing communication & collaboration in interdisciplinary research: sage publications.

[72] Norton SA, Wittink MN, Duberstein PR, Prigerson HG, Stanek S, Epstein RM (2019). Family caregiver descriptions of stopping chemotherapy and end-of-life transitions. Supportive Care Cancer.

[73] Johnson CE, Girgis A, Paul CL, Currow DC (2008). Cancer specialists' palliative care referral practices and perceptions: results of a national survey. Palliat Med.

[74] Johnson C, Paul C, Girgis A, Adams J, Currow DC (2011). Australian general practitioners’ and oncology specialists’ perceptions of barriers and facilitators of access to specialist palliative care services. J Palliat Med.

[75] Le B, Mileshkin L, Doan K, Saward D, Spruyt O, Yoong J, Gunawardana D, Conron M, Philip J (2014). Acceptability of early integration of palliative care in patients with incurable lung cancer. J Palliat Med.

[76] Broom A, Kirby E (2013). The end of life and the family: hospice patients’ views on dying as relational. Sociol Health Illness.

[77] Le BHC, Mileshkin L, Doan K, Saward D, Spruyt O, Yoong J, Gunawardana D, Conron M, Philip J (2014). Acceptability of early integration of palliative care in patients with incurable lung cancer. J Palliat Med.

[78] Groot MM, Vernooij-Dassen MJFJ, Verhagen SCA, Crul BJP, Grol RPTM (2007). Obstacles to the delivery of primary palliative care as perceived by GPs. Palliat Med.

[79] Smith CB, Nelson JE, Berman AR, Powell CA, Fleischman J, Salazar-Schicchi J, Wisnivesky JP (2012). Lung cancer physicians' referral practices for palliative care consultation. Ann Oncol.

[80] Appleton L, Poole H, Wall C (2018). Being in safe hands: Patients' perceptions of how cancer services may support psychological well-being. J Adv Nurs.

[81] Hovland CA, Kramer BJ (2019). Barriers and facilitators to preparedness for death: experiences of family caregivers of elders with dementia. J Soc Work End Life Palliat Care.

[82] El-Jawahri A, LeBlanc TW, Burns LJ, Denzen E, Meyer C, Mau LW, Roeland EJ, Wood WA, Petersdorf E (2018). What do transplant physicians think about palliative care? A national survey study. Cancer.

[83] Balshem H, Helfand M, Schünemann HJ, Oxman AD, Kunz R, Brozek J, Vist GE, Falck-Ytter Y, Meerpohl J, Norris S (2011). GRADE guidelines: 3. Rating the quality of evidence. J Clin Epidemiol.

[84] Kolben T, Haberland B, Degenhardt T, Burgmann M, Koenig A, Kolben TM, Ulbach K, Mahner S, Bausewein C, Harbeck N (2018). Evaluation of an interdisciplinary palliative care inhouse training for professionals in gynecological oncology. Arch Gynecol Obstetr.

[85] Lam P-L, Lam T-C, Choi C-W, Lee AW-M, Yuen K-K, Leung T-W (2018). The impact of palliative care training for oncologists and integrative palliative service in a public-funded hospital cluster-a retrospective cohort study. Support Care Cancer.

[86] Ahaddour C, Van den Branden S, Broeckaert B (2018). Between quality of life and hope. Attitudes and beliefs of Muslim women toward withholding and withdrawing life-sustaining treatments. Med Health Care Philos.

[87] Chung BPM, Leung D, Leung SM, Loke AY (2018). Beyond death and dying: how Chinese spouses navigate the final days with their loved ones suffering from terminal cancer. Support Care Cancer.

[88] Grant R, Finocchio L (1995). Interdisciplinary collaborative teams in primary care: a model curriculum and resource guide: pew health professions commission.

[89] Bradley EH, Cramer LD, Bogardus ST, Kasl SV, Johns on-Hurzeler R, Horwitz SM (2002). Physicians' ratings of their knowledge, attitudes, and end-of-life-care practices. Acad Med.

[90] Redman S, White K, Ryan E, Hennrikus D (1995). Professional needs of palliative care nurses in New South Wales. Palliat Med.

[91] Walshe C, Chew-Graham C, Todd C, Caress A (2008). What in fluences referrals within community palliative care services? A qualitative case study. Soc Sci Med.

