# Supplementary material for: Palliative care utilization in oncology and hemato-oncology: a systematic review of cognitive barriers and facilitators from the perspective of healthcare professionals, adult patients, and their families
Source: BMC Palliat Care. 2020 Apr 13;19:47. doi: 10.1186/s12904-020-00556-7 (PMC7155286; doi:10.1186/s12904-020-00556-7)
Supplement: Supplementary file 1 — Additional file 1:. [file 12904_2020_556_MOESM1_ESM.docx]

**Appendix A:**

**Search terms**

**SEARCH 1: Pubmed**

1. (((((((palliative care OR palliative medicine OR palliative treatment OR palliative therapy OR supportive care OR supportive medicine OR supportive treatment OR supportive therapy OR end of life care OR end of life medicine OR end of life treatment OR end of life therapy OR terminal care OR terminal medicine OR terminal treatment OR terminal therapy OR hospice care OR hospice medicine OR hospice treatment OR hospice therapy OR Hospice*[MeSH])) AND
2. (Facilitat* OR Enabl* OR Encourag* OR Support* OR Cause* OR Barrier* OR Obstacl* OR Hurdl* OR Barricad* OR Obstruct* OR Discourag*[MeSH]))

AND

(Attitud* OR Point* of view OR Perspective* OR Angl* OR Position* OR Thought* OR Belief* OR Idea*[MeSH Terms])))))

AND

1. (Humans[Mesh] AND English[lang] AND adult[MeSH])

Limits: Humans, Adults (19 and older), English.

Results: 5,201 (06.06.2019) (before deleting duplicates)

**SEARCH 2: PsychINFO**

1. SU ( Palliative care OR palliative medicine OR palliative treatment OR palliative therapy OR supportive care OR supportive medicine OR supportive treatment OR supportive therapy OR end of life care OR end of life medicine OR end of life treatment OR end of life therapy OR terminal care OR terminal medicine OR terminal treatment OR terminal therapy OR hospice care OR hospice medicine OR hospice treatment OR hospice therapy OR Hospice* ) AND
2. TX ( Facilitat* OR Enabl* OR Encourag* OR Support* OR Cause* OR Barrier* OR Obstacl* OR Hurdl* OR Barricad* OR Obstruct* OR Discourag* )

AND

1. TX ( Attitud* OR Point* of view OR Perspective* OR Angl* OR Position* OR Thought* OR Belief* OR Idea* )

Limits: Adults (18 and older) and language English.

Results: 2,107 (01.06.2019) (before deleting duplicates)

**SEARCH 3: IAHPC** (International Association for Hospice and Palliative Care**/CINAHL** (Nursing journal databases)

1. SU ( Palliative care OR palliative medicine OR palliative treatment OR palliative therapy OR supportive care OR supportive medicine OR supportive treatment OR supportive therapy OR end of life care OR end of life medicine OR end of life treatment OR end of life therapy OR terminal care OR terminal medicine OR terminal treatment OR terminal therapy OR hospice care OR hospice medicine OR hospice treatment OR hospice therapy OR Hospice* )

AND

1. TX ( Facilitat* OR Enabl* OR Encourag* OR Support* OR Cause* OR Barrier* OR Obstacl* OR Hurdl* OR Barricad* OR Obstruct* OR Discourag* )

AND

1. TX ( Attitud* OR Point* of view OR Perspective* OR Angl* OR Position* OR Thought* OR Belief* OR Idea* )

Limits: All adults and language English.

Results: 1,989 (07.06.2019) (before deleting duplicates)

**SEARCH 4: Communication and mass media complete**

1. SU ( Palliative care OR palliative medicine OR palliative treatment OR palliative therapy OR supportive care OR supportive medicine OR supportive treatment OR supportive therapy OR end of life care OR end of life medicine OR end of life treatment OR end of life therapy OR terminal care OR terminal medicine OR terminal treatment OR terminal therapy OR hospice care OR hospice medicine OR hospice treatment OR hospice therapy OR Hospice* )

AND

1. TX ( Facilitat* OR Enabl* OR Encourag* OR Support* OR Cause* OR Barrier* OR Obstacl* OR Hurdl* OR Barricad* OR Obstruct* OR Discourag* )

AND

1. TX ( Attitud* OR Point* of view OR Perspective* OR Angl* OR Position* OR Thought* OR Belief* OR Idea*)

Limits: All adults and language English.

Results: 114 (07.06.2019) (before deleting duplicates)
