# Supplementary material for: Palliative care utilization in oncology and hemato-oncology: a systematic review of cognitive barriers and facilitators from the perspective of healthcare professionals, adult patients, and their families
Source: BMC Palliat Care. 2020 Apr 13;19:47. doi: 10.1186/s12904-020-00556-7 (PMC7155286; doi:10.1186/s12904-020-00556-7)
Supplement: Supplementary file 2 — Additional file 2:. [file 12904_2020_556_MOESM2_ESM.docx]

**Appendix B**

**Table 3 - Summarized results of the selected studies**

| ***Study no.** | **Author/s and year** | **Barriers** | **Facilitators** | **Proposed strategies** | **Theme/s related to** | **Outcome/s** |
| --- | --- | --- | --- | --- | --- | --- |
| 1 | Akiyama, M., Takebayashi, T., et al., 2012 | Beliefs that PC is only for terminally ill patients. | n/a | To promote patients’ sense of security strategies proposed are providing correct information about the safety of opioids, the availability of PC during the entire course of the disease, and about homecare characteristics. | Attitudes and beliefs | Quality PC provision |
| 2 | Alaeddini, J., Julliard, K., et al., 2000 | Lack of PC education for physicians, residents, other HPs and the general public.  Lack of hospital support systems to implement PC appropriately, and a lack of knowledge and support regarding legal considerations. No clear guidelines on these issues. | Availability of PC inter-disciplinary units where diverse professionals (e.g. nurses, social workers, physicians specialists in PC) collaborate.  PC team paying attention to ethical differences and acting according to patient values. | Creating PC units which could be cost-effective, bring comfort to patients and contribute preventing patients from suffering non-necessary interventions. | Awareness | Quality end-of- life care provision and PC implementation |
| 3 | Ansari, M., Rassouli, M.,  et al., 2018 | Related to health care providers (HPs): Lack of education in PC. Lack of understanding on what PC consist in. Lack of standard educational content for PC at universities. Lack of familiarity with the basic principles of PC among students (medical disciplines). Lack of practical education program on PC.  Related to specific aspects regarding HPs-family/patients: Lack of information provided to patients. Lack of public awareness about available PC services. Lack of attention to patients' customs. Lack of attention to patients' beliefs. Lack of attention to patients' culture. Lack of communication skills. Lack of familiarity with the basic principles of PC among the general population/patients/families. Stigma of cancer associated to PC. Lack of guidelines for providing patient education. Lack of specific educational programs for caregivers. Absence of means of communication for patients/families with the relevant healthcare centers. | Motivation in HPs in providing effective care.  Patients/families desire to receive information from reliable sources. | Meeting the educational needs of the stakeholders of palliative care is urget and requires policymakers  to identify the aspects leading to strategies based on the use of opportunities, coping with the threats to which the organization is faced and the removal of weaknesses. | Awareness  Collaboration and communication  Attitudes and beliefs | Palliative care educational needs |
| 4 | Beernaert, K., Deliens, L., et al., 2014 | Barriers were related to communication styles, the perceived role of a family physician (FP) and continuity of care.  Scarce communication on PC needs between patients and FP (who are more perceived as the person to appeal to in acute and standard follow-up situations).  FP pay more attention to PC needs in the patient terminal phase.  FP lack of knowledge of, and skills and experience about talking of, existential needs. FP difficulty seeing patient dying.  Patient lost ability to communicate.  FP time constraints. Late involvement of nurses by FP. FP not using communication tools. FP not participating in multidisciplinary meetings. FP not seeing the patient during treatment phase. No patient hospital medical files transfer to FP. FP concerns openly communicating medical condition takes away hope of patient/family. | Effective communication processes.  Proactive questioning about care needs.  Gentle exploration.  The use of short assessment scales, communication note-books and medical files with non-physical information.  Patient openness about care needs.  Patient writing questions and needs on a paper.  FP creating a sense of trust in the patient.  FP making regular contact (visit, phone) with the patient.  FP making regular home visits.  FP paying attention to nonphysical needs.  FP as being the person to whom you can say anything.  FP contacting the patient during treatment phase.  Nurses informing the FP.  FP using communication tools.  Medical files including nonphysical issues.  Family telling care needs to FP.  FP having features typically  related to women (e.g.  empathy).  FP realizing that the patient suffering from a life-threatening disease.  FP recognizing when the patient is in the terminal phase. | The use of proactive communication and communication tools could contribute to the development of guidelines for family physicians and policymakers in primary care in order to improve the early identification of PC needs. | Awareness  Attitudes and beliefs  Collaboration and communication | Early identification of PC needs |
| 5 | Boyd, D., Merkh, K., et al. , 2011 | Lack of confidence talking about death with patients and families among physicians (74% of the sample agree) or among nurses (23% agree).  Type of role and tasks of nurses not allowing raising hospice as an option without prior physician discussion.  Nurses perceptions that discussing the chance of the hospice leads to loss of hope for patients.  Lack of patient or family member PC acceptance.  Physician reluctance (in 4/10) to refer patients to hospice or PC. | Having had a previous experience and previous referrals to hospice. Receiving hospice trainings.  Positive attitudes towards end-of-life practices and PC use.  Non experiencing discomfort discussing prognosis. Recognizing the importance of discussing prognosis. | Strategies should be developed to enable nurses to have a stronger voice during this critical time for patients. Further education should be provided to them, including trainings to improve their capacity to deliver effective interventions to terminally ill patients with cancer. | Awareness  Attitudes and beliefs  Collaboration and communication | End-of-life options’ discussion and hospice referral |
| 6 | Bradley, E. H., Cramer, L. D., et al., 2002 | n/a | Recognizing the importance of discussing prognosis. Having knowledge about terminal care.  Positive attitudes towards palliative philosophy and practices.  Having had a previous experience in referral for hospice care. | Medical school curricula and continuing education programs should be developed to better cover the relevant aspects of caring for the dying. | Awareness  Attitudes and beliefs | PC referral |
| 7 | Broom, A., Kirby, E. et al., 2012 | Communication issues involving the patient and family care giver and the specialist (e.g. avoiding uncomfortable conversations about death and dying). Difficulty in handing over/‘letting go’ of patients. Physician personal crusades against diseases. Inter-professional variation.  Lacking inter-specialty communication and cross-disciplinary dialogue. Patient or family care giver resistance/denial. Lack of onsite PC services. Difficulty navigating bureaucracy. | n/a | To improve patient care quality or timely referral it is relevant to develop streamlined practices that are sensitive to specialty needs and patient desires, with the focus on the inter-specialty dialogue. | Awareness  Attitudes and beliefs  Collaboration and communication  Emotions | Timely PC referral |
| 8 | Canzona, M. R., Love, D., et al., 2018 | Barriers among nurses:  Difficulty in coping with interprofessional communication errors during the transition to PC.  Difficulty in responding to patient/family reactions to miscommunication about the goals of care.  Struggling to determine the appropriate step when misperceptions (in patients/family) are discovered.  Lack of information provided by other providers (other than nurses) about changes in patient prognosis.  Difficulty in dealing with conflicting information about the goals of care which are them given.  Difficulty in adapting to sociocultural factors that influence information.  Difficulty in navigating emotional connection when this is influenced by sociocultural factors when proving information on health condition.  Difficulty in responding to patient shock.  Difficulty in responding to patient anger.  Difficulty in responding to patient despair.  Difficulty in responding to patient denial.  Difficulty in processing their own emotions about patient prognosis.  Over-identifying with patients and families.  Difficulty in adapting to patients’ cultural norms.  Difficulty in adapting to patients’ religious beliefs.  Difficulty in communicating with families in conflict. | Nurses education including emotion management and communication skills. | To reduce nurses’ emotional burden, and protect  suffering patients and families from additional distress, strategies should include a narrative-based approaches to education and  practice for nurses working in cancer care. | Collaboration and communication  Emotions  Attitudes and beliefs | Transition to palliative care |
| 9 | Cherny, N. I. and Catane, R., 2003 | Lack of adequate training in PC.  Lack of trust in PC specialists regarding understanding of oncological area/treatments.  No clear guide on PC coordination roles (for patients with advanced cancer).  Negative views regarding oncologists’ involvement in PC (in 15% of the sample). | n/a | To create infrastructural changes to facilitate better actualization of PC in clinical practice for patients with advanced cancer. In particular, creating and developing comprehensive cancer centers and community hospitals.  To increase the recognition of the importance of PC and supportive care for patient with advance cancer among oncologists and an adequate preparation to coordinate the end-of-life care for their patients. | Awareness  Attitudes and beliefs  Collaboration and communication | Optimal supportive and PC provision |
| 10 | Feeg, V. D. and Elebiary, H., 2005 | Patient family unwillingness to refer the patient to PC.  Mandatory rule for the patient to be first declared dying or terminal to qualify for specialized hospice care.  Physician reluctance to make referral.  Physician lack of familiarity with availability and suitability of hospice, therefore, without he/she cannot access to PC.  Association of hospice with death.  Lacking information about severity of illness.  Lacking information about irreversibility of illness. | n/a | Strategies aiming to improving and expand hospice and PC services despite the system, structural, financial, or perceived obstacles should applied. | Awareness  Attitudes and beliefs | Hospice and PC access |
| 11 | Fox, J., Windsor, C. et al., 2016 | Uncertain scope of PC for users and for those working in the health care system.  Difficulties in negotiating the transition to PC, due to the ambiguity and tension around referral. | n/a | A major strategy would include achieving greater coherency of care within an increasingly specialized healthcare system. | Awareness  Collaboration and communication | Transition to the PC process |
| 12 | Gidwani, R., Nevedal, A., et al., 2017 | Resource constraints (PC provided only at end of life).  Non homogenous perspective on primary versus specialist PC.  Under-availability of outpatient PC.  Poor communication about prognosis and care plans among oncologists and PC specialists.  Perception of PC as a ‘‘team of outsiders’’.  Perception of the focus of PC as too narrow. | Improvement of the interface between oncologists and PC providers. In particular, definition of a clear division of responsibility, encouragement in-person collaboration and “sharing” of non-physician palliative team members. | Strategies should include mitigation conflicting prognoses and care practices in inpatient PC. This could be achieved through increased availability and use of outpatient PC. | Awareness  Attitudes and beliefs  Collaboration and communication | PC provision |
| 13 | Gott, M., Ingleton, C. et al., 2011 | Lack of routine discussion of prognosis with inpatients. Lack of discussion about adopting PC approach to patient management with patients (these were often discharged from hospital with “false hope” of cure).  Lack of effective communication between health professionals and patients. | n/a | Strategies proposed include education and training for generalist PC providers (tailored specifically to the unique nature of acute hospital settings).  Further research on, for instance, interventions that enable all hospital team members to signal a potential transition to palliative care or that encourage communication between hospital teams. | Collaboration and communication | Transition to the PC process |
| 14 | Groot, M. M., Vernooij-Dassen, M. J. et al., 2005 | Personal level barriers: inadequate knowledge and expertise, emotional issues related to role and responsibility of providing PC. Insufficient coordination and collaboration among HPs.  Relational level: communication problems with patient/relative and communication with other HPs (e.g. difficult to assess whether patients have informed them about their problems and needs). | n/a | A major suggested strategy consists in helping general practitioners acquire the appropriate balance between technical and organizational interventions and a compassionate orientation to their terminally ill patients. | Awareness  Collaboration and communication | PC task performance |
| 15 | Miyashita, M., Hirai, K. et al., 2008 | A negative image of PC units by patients and families.  Delay of termination of anti-cancer treatment by physicians in the general wards.  Unwillingness to end anti-cancer  treatment and denial of the fatal nature of the disease by  patients and families.  Patient’s wish to receive care from familiar physicians and nurses.  Insufficient knowledge of PC units by medical staff in general wards. | n/a | Strategies include to correct these unfavorable images and misconceptions of PC units, and facilitate an early introduction of PC options to patients, finally, to provide communication skill training regarding breaking bad news to HPs. | Collaboration and communication  Awareness  Attitudes and beliefs | Referral to inpatient PC unit |
| 16 | Horlait, M., Chambaere, K. et al., 2016 | Oncologist-related barriers to discuss PC: emotional bond, emotional discomfort, lack of training in communication skills, and lack of experience.  Patient-related barriers to discuss PC: language/culture barriers, medical co-morbidities, insufficient level of intelligence for adequate level of communication. Additionally, barriers related to emotions in patients such as sadness/suppression, unrealistic expectations, infinite trust and faith in medicine, giving up hope, overly optimistic about life expectation.  Family-related barriers to discuss PC: protection of the patient, family disputes, unrealistic expectations, emotional reactions.  Barriers related to the referring physician to discuss PC: lack of guidelines/protocol, late referral, and reluctance to discuss disease stage.  Barriers related to disease and treatment: patient physical condition, unpredictable trajectory and inability to estimate prognosis.  Other barriers: issues related to characteristics of the setting, to team dynamics and responsibilities, to practical organization, to workload or to lacking support and coaching.  Societal and policy barriers: PC stigma and lack of integration of PC in medical education, and predominance of curative model.  No formal training in communication skills in medical training.  No formal training in medical training.  No PC service or delay in availability of PC service. | n/a | These barriers should serve as a  starting point for revising the medical education of oncologists.  Additionally, these barriers should be taken into consideration by hospital management and policy makers to be aware of their impact on the daily practice of oncology and finally to deploy all possible actions in order to improve PC access. | Emotions  Attitudes and beliefs | Referral to inpatient PC unit |
| 17 | Hui, D., Cerana, M. A. et al., 2016 | Barriers for hematologic oncology specialists lower comfort levels towards PC referral (compared to solid tumor specialists).  Being less comfortable with end-of-life care. | Increasing comfort with symptom management.  Increasing provision of counselling.  Being trained both in oncology and PC. | Strategies should include support and education for those oncologists who are less comfortable with end-of-life care. | Awareness  Collaboration and communication | Primary PC provision and specialized PC referral |
| 18 | Hui, D., Park, M. et al., 2015 | Being hematologic specialists (these were less likely to refer patients early in the disease trajectory to PC specialists).  They were more conducive to referral with the service name supportive care instead of PC.  The use of the term PC (compare to the use of the term supportive care). | Using the term supportive care instead of PC. | A major strategy consists in rebrand palliative care to overcome the stigma associated with this term and this may improve patient access to PC services. | Collaboration and communication  Attitudes and beliefs | General PC provision and specialized PC referral |
| 19 | Johnson, C., Paul, C. et al., 2010 | Lack of ability to communicate openly and honestly about disease progression by health professionals.  Divergent view about appropriate timing for PC access.  Predominant view about specialized PC services (dedicated to physical symptoms management, not for those who have complex problems without physical symptoms). | n/a | Strategies should focus on objectively identifying patients with greatest need of specialized PC services across all domains of care and these should be applied regardless of timing, stage of disease, or type of treatment. | Collaboration and communication  Attitudes and beliefs | PC referral |
| 20 | Kafadar, D., Ince, N. et al., 2015 | Lack of interest on and knowledge of PC among HPs.  Belief that PC is not appropriate for those who have complex problems without physical symptoms.  Absence of awareness of PC or specific cultural beliefs among patient families. | Providing education for HPs and patient families.  Raising awareness in society. | Strategies should include increasing the capacity of health professionals to provide care in every stage of cancer. These strategies should also consider effective education planning and patient care management. | Awareness  Collaboration and communication | PC provision and integrating into health care system |
| 21 | Kawaguchi, S., Mirza, R. et al., 2017 | Lacking knowledge in providing PC in practice (how to  bridge that gap between theory and practice).  Misconceptions about PC patients and families.  Conflicts between patients and their family.  Lack of knowledge on how/when initiate PC. Lack of knowledge in communicating effectively with patients and families and properly managing patients’ symptoms. Lack of understanding available resources. | Knowing how and when to initiate a palliative approach.  Improving communication skills.  Improving symptom management skills.  Identifying available resources.  Recognizing the importance of PC.  Improving education on PC in general, including experiencing observation and receiving support. | Strategies should consider incorporating supplement experiential learning on palliative care to the training programs. Additionally, clinicians can further improved learning by informal teaching and direct observation, and identifying themselves as resources for learners. A further strategy includes facilitate partnerships between internal medicine and PC programs. | Awareness  Collaboration and communication  Attitudes and beliefs  Emotions | Ability to provide PC |
| 22 | Keim-Malpass, J., Mitchell, E. M. et al., 2015 | Unclear pathways and triggers for referral.  Demand exceeding available practitioners.  Insufficient or inadequate education for patients and oncology providers on PC and hospice care | n/a | Strategies aiming to improve integration of PC should consider aspects from a process, education and system perspective. | Awareness  Collaboration and communication | PC integration |
| 23 | Kirby, E., Broom, A. et al., 2014 | n/a | Family into care trajectories and decision-making inclusion.  Patient family involvement can have a relevant role in helping persuade the patient to begin specialist PC (e.g. through translating medical language or reinforcing specialists’ opinions and proposed treatment and transition). | Possible strategies should consider the centrality of families in key moments in care and how these can be managed by those HPs involved in the patient treatment and in PC services. | Collaboration and communication | Transition to specialized PC |
| 24 | Kumar, P., Casarett, D. et al., 2012 | Having lung cancer (as this condition was associated with less specialized PC services, compared to those having breast cancer).  Lack of awareness about PC in patients.  Lack of physician referral. | Patient (general) graduate education (associated with greater supportive and PC service use). | A major strategies would be further research on understanding patients’ needs and beliefs regarding PC services and how to integrate these into conventional treatments with the aim to improve cancer care. | Awareness  Attitudes and beliefs | To access to PC services |
| 25 | Le, B. H., Mileshkin, C., L. et al., 2013 | Lacking competence/skills on quality PC and capability of PC provision.  Lacking care coordination (needed to ensure integrated care).  Belief that early PC introduction brings confusion over roles, and increases care fragmentation.  Lacking team communication skills.  Belief that potentially reversible conditions are missed when using PC.  Non ease of referral (needed for ready access to a PC provider in the lung cancer clinic).  Beliefs about loss of hope and fears of negative patient reaction when PC is introduced.  PC term negatively perceived.  Beliefs that patient or family might have inappropriate reactions to referrals among clinicians. | n/a | Strategies aiming at doing the PC team a front-line team members should consider education for lung cancer clinicians about the role and benefits of early PC, and how best to introduce it. | Awareness  Attitudes and beliefs | Implementation of early referral to PC |
| 26 | Le, B. H. C. and Watt, J. N., 2010 | Uncertainty about the role of PC. Discrepancy between medical and nursing views of the utility and timing for PC. | Referral for PC consultations was associated with:  - Improvements in availability of appropriate  end-of-life medication orders, communication with patients and families.  - Cessation of futile treatment and interventions. | Strategies should consider the need for more frequent and/or earlier referral of dying patients and the need for generalist staff to become more skilled in the delivery of PC. These should also take into account the need for PC clinicians to be educators and advocates within services to enhance care at end-of-life. | Awareness  Collaboration and communication | PC referral |
| 27 | LeBlanc, T. W., O'Donnell, J. D. et al., 2015 | For most hematologic oncologists:  Perception of palliative care as end-of-life care.  Control/distrust of other and their oncology knowledge.  Different treatment goals, responsiveness to chemotherapy  Preference to maintain the control even palliative aspects of patient care.  For most solid tumor oncologists:  Perception of PC as a subspecialty that could assist with complex patient cases.  Low awareness of available services. | n/a | Effective strategies targeting PC integration into hematologic malignancy practices should address unique barriers to PC referral experienced by hematologic malignancy specialists and, therefore, be tailored to them. | Awareness  Attitudes and beliefs | PC access |
| 28 | Llamas, K. J., Llamas, M. et al., 2001 | Over-emphasis on active treatment of cancer.  Lack of or inappropriate palliative education of staff.  Non addressed the need for improved staff support.  Problems with co-ordination of care.  Communication problems.  Staff attitudes.  Lack of spiritual support for patients and families.  Lack of outreach PC support service for patients/families after discharge.  Lack of staff support.  Violations of patient autonomy.  Lack of community supports.  Lack of information regarding available supports/services. | n/a | Strategies should address the need for a designated PC service in order to improve the standard of care of dying cancer patients, as well as the staff need for improved PC education and support. | Awareness  Attitudes and beliefs  Collaboration and communication | PC delivery |
| 29 | Mahon, M. M. and McAuley, W. J., 2010 | Incomplete or incorrect personal understandings of PC.  Perception of PC as mostly only focused on symptom management.  Lack of knowledge of PC and hospice, and of these services’ aims.  Perception of their own role in decisions regarding PC to be limited and indirect. | Broader education about clinical experience with PC. | Strategies aiming to enhance nurses’ education and clinical experience in a continuous quality-improvement model are needed in order to overcome barriers to appropriate PC provision. | Awareness  Attitudes and beliefs | PC program implementation |
| 30 | McDarby, M. and Carpenter, B. D., 2019 | Barriers related to interaction between providers:  Lack of interactions between health providers (cancer care and PC);  Lack of opportunity for conversations between providers;  Informal interactions focused on patient care (no additional topics);  Providers only professional relationships with one another.  Barriers related to patient and family perceptions about palliative care:  Resistance from patients and their family to the involvement from the PC team;  Patient and families do not know the purpose of PC involvement.  Barriers related to provider attitudes toward palliative:  Beliefs that provider can provide PC himself;  Belief PC team consultation means failure;  Belief PC team consultation equals to last resort.  Barriers related to education and training about palliative care:  Lack of PC in curricula;  Lack of practical education program on PC;  Lack of knowledge of the meaning of PC and hospice care.  Barriers related to the role of the palliative consultation team:  Providers are hesitant about some medications;  Divergent expactations about communication role of PC team.  Barriers related to palliative care consultation team recommendation  implementation:  Providers are hesitant about prescribing some medications. | Facilitators related to interactions between providers:  PC team strategic visibility in patient floors and hospital-wide events;  PC team unintentional visibility like being present around the hospital;  PC team nurturing casual relationships.  Facilitators related to patient and family perceptions about palliative care:  Increase of opportunities of patient education for instance videos;  Education of community;  Bolster support through positive outpatient experiences.  Facilitators related to provider attitudes toward palliative care:  Demonstration of the utility of PC for patients via exposure;  Cultivation of trusted relationships with providers who may consult.  Facilitators related to education and training about palliative care:  Provision of education to providers earlier in their training;  Demonstration of the utility of PC across disciplines.  Facilitators related to the role of the palliative care consultation team:  Marketing of the role of PC team;  Provision of education and scope of competencies.  Facilitators related to palliative care consultation team recommendation implementation:  Provision of clear, convincing, scientific support for recommendations;  Use of verb ‘recommend’ instead of ‘suggest’ for recommendations;  Provision of a follow-up with direct contact to explain recommendations;  During follow-up explain the state requiring an urgent implementation. | Strategies aiming at an effective collaboration with other specialty providers, the palliative care consultation team may consider strategies including structured educational interventions, increased visibility in the hospital, and active marketing of the utility and relevance of palliative care across disciplines. | Collaboration and communication  Awareness  Emotions | To hinder palliative care consultation |
| 31 | McGrath, P., 2013 | Among hemato-oncology patients:  No will to discuss end-of-life or will to discuss it later.  Preference to talk about PC when it is necessary for an immediate referral (barriers to timely referral).  Beliefs that discussing PC at the point of diagnosis and in early treatment is wrong (there is already too much to think about). | Knowledge PC benefits. | Strategies should include an active role of social workers in educating, supporting, advocating and referring hematology patients in order to ensure that appropriately timed involvement in PC will occur. | Attitudes and beliefs | PC access (onco-hematology) |
| 32 | McIlfatrick, S., 2007 | Difficulty in defining the term PC. No consensus of the right timing to initiate PC among HPs.  Difficulty in communication and information exchange during the discharge from acute to community care.  Breakdown in communication. | n/a | Strategies aiming to guarantee access to PC regardless of diagnosis should consider an integrated approach to PC. | Awareness  Attitudes and beliefs  Collaboration and communication | PC access |
| 33 | Melvin, C. S., 2010 | Lack of knowledge about PC services and its benefits.  Need for medical referral to access PC.  Beliefs that PC should be provided in the last 48/72 hours before death. | Receiving an accurate estimate of survival by HPs. | Strategies should consider education about PC /hospice care services to be provided both to general practitioners as well as to the general public to empower them to seek out these services as needs arise. | Awareness  Attitudes and beliefs | Early PC referral |
| 34 | Mohammed, S., Swami, N., 2018 | Difficulty in navigating the home care system to access resources for caregivers. | Engaging and interacting with visiting health care professionals (HPs).  As caregiver being described as of value as the HPs providing care at home.  Provision of detailed explanations about what to expect at the end of life from HPs. | Policies, quality indicators, and guidelines should ensure the provision of comprehensive, interdisciplinary home palliative care. | Awareness  Collaboration and communication | Quality cancer patients at home receiving |
| 35 | Monterosso, L., Ross-Adjie, G. M. et al., 2016 | Lacking understanding of the broad applicability of PC among HPs.  Misconceptions about PC and its services among HPs.  Insufficient awareness of end of life care (e.g. beliefs about mutual exclusion of PC and medical cancer treatment) among families.  Lacking education on PC practice among HPs. | Increasing awareness among patients and families about hospice referral. Inform that this does not necessarily mean end of life but could include effective interventions for symptom control allowing discharge home.  Offering training on PC to HPs (e.g. PC practice through ‘shadowing’ and ‘mentoring’ by hospice PC staff, workshop) and development of area-specific education and clinical guidelines on PC. | Strategies should take into account an innovative approach to education and upskilling HPs in PC and referral pathways. | Awareness  Collaboration and communication  Attitudes and beliefs | PC provision |
| 36 | Norton, S. A., Wittink, M. N., et al., 2018 | No shared understanding about prognosis and treatment choices between health care professional and patients;  disconnects between patients, caregivers, oncologists;  patient will to continue active treatment despite its limited effectiveness;  caregivers and patients are aware of incurability of cancer but unaware of the nearness of death;  transitions to comfort care are chaotic (according to patient/family perception);  transitions to comfort care within days of death or did not occur;  lack of shared prospectives communicated;  late transition to comfort care;  caregivers are unprepared for rapid deteriorations of patient conditions;  oncologists were not responsive, as caregivers would have liked, when the deteriorations of patient conditions rapidly worsen;  no end of life discussions with oncologists;  caregivers felt abandoned by oncology team;  lack of clarity of which health care professional caregivers should call;  lack of recognition that patient's deteriorating condition indicated imminent death;  caregivers interpretations of oncologists' communication reinforced hope patient condition would considerably improve caregivers after this first interpretation felt abandoned;  no discussions about prognosis with caregivers;  no discussions about time transitions to comfort-oriented care;  no consistency between oncologists and emergency department team on patient life-timing expectations;  no consistency between caregivers assumptions about patients' illness trajectories and actual patients' conditions;  caregivers realized actual conditions felt frustrated and angry;  some caregivers were excluded from patient-oncologist conversations even from end-of-life conversations;  caregivers perception of a disturbing liminal state between active cancer treatment and end of life (feeling of abandonment);  no appropriate preparation of the caregivers to the transition by health care professionals, only when crisis of patient's condition occurred;  in the patient crisis transition caregivers (unprepared) experienced several negative feelings (e.g. trauma, distress, anxiety, panic, sense of abandonment);  no enough information prior to the crisis transition pattern are given to patients and their family;  decisions about life-sustaining are made hastily, these are requested to be made too quickly;  decisions about life-sustaining requested to patients/family cannot be made with the involvement of the known physicians who they know. | n/a | The results highlight the need to more effective conversations between clinicians and patients regarding end-of-life choices for patients with advanced cancer. Future strategies should take into consideration the different patterns of transition and their unique patient and caregiver needs. Additionally, these strategies should take into account that variation in transitions from active treatment to death are considerable, and potentially unwarranted. | Collaboration and communication  Emotions  Attitudes and beliefs | End-of-life transition  (perceptions of caregivers) |
| 37 | O'Connor, M. and Lee-Steere, R., 2006 | Difficulty in discussing spiritual issues with patients, such as death and dying. Difficulty in talking about psychosocial and emotional problems such as depression or difficulties accepting what was happening among patients.  Uncomfortable with proposing PC to new patients (no continuity in providing PC).  Lacking support regarding the emotional involvement with the patient and family.  Difficulty addressing patient family reaction to prognosis disclosure.  Beliefs about loosing the main control of patient care among GPs. | Further education and training focused on dealing properly with patient psychosocial and emotional problems for HPs. | Strategies to overcome the barriers to PC provision should include establishing multidisciplinary PC team and continuing education in pain and symptom control and communication. | Awareness  Attitudes and beliefs  Collaboration and communication  Emotions | PC provision |
| 38 | Odejide, D. Y. Salas Coronado, et al., 2014 | Being a young patient.  Difficulty to identify the end of life phase in hematology cancer (due to the nature of cancer and continuing potential for cure with advanced disease).  Unrealistic expectations from both physicians and patients.  Long-term patient-physician relationships resulting in difficulty conducting end of life discussions.  Inadequacy of existing home-based end-of-life services.  Beliefs that end-of-life measures are specifically developed for solid tumor cancer rather than for blood cancer. | n/a | Effective strategies should include the provision of clinical markers for when to initiate end-of-life care to hematologic oncologists. | Attitudes and beliefs | End-of-life care initiation |
| 39 | Patel, M. I., Periyakoil, V. S., 2018 | Health care professionals (HPs)’ difficulty in communicating patients' prognoses  HPs interchanging palliative care and hospice services.  HPs feeling of being compelled to fully exhaust disease-directed treatment options prior to referring patients to palliative care specialists.  HPs being fearful of providing insufficient treatment.  When HPs referred to palliative care, some patients voiced dissatisfaction, noting unmet expectations. | Integration of a health coach to assist HPs with patient education and with caregivers in advance care planning.  Designation of proactive approaches for non-face-to-face communication with patients and family. | Strategies to improve cancer care at end of life need to take into account that providers’ perspectives are critical to  redesigning care delivery such that it is effective and more  patient centered. Successful implementation of these  approaches depends massively on the input and acceptance of  health-care professionals involved in the delivery of care at the end of life. | Awareness  Collaboration and communication  Attitudes and beliefs | End-of-life cancer care  provision |
| 40 | Philip, J. A. M. and Komesaroff, P., 2006 | Family-related barriers: Lack of openness of communication, particularly within families. Forbidding discussion of both diagnosis and prognosis.  Health care professional-related barriers: Lack of clear communication between health professional and patient/family. Lack of knowledge about PC options. Personal needs of the health professional.  Patient will to stay at home. | n/a | Strategies to pursue ideal care should consider a complex mix of aspirations, real world practicalities and fulfilment for both patients and staff. | Awareness  Attitudes and beliefs | PC access |
| 41 | Redman, S., White, K. et al., 1995 | Lack of opportunity for formal study.  Insufficient clinical knowledge. | n/a | Strategies should comprise training and other professional support to PC nurses. | Awareness | PC provision |
| 42 | Rhee, J. J.-O., Zwar, N. et al., 2008 | Lacking interest and knowledge in PC among GP.  Limited confidence about psychosocial problems.  Lack of confidence about technical aspects. | n/a | Strategies which is intended to increase general practitioners’ involvement in PC aim at increasing their knowledge and interest in PC. Additionally, innovations in service provider models may contribute to overcome the barriers to provision of after-hours care. | Awareness  Attitudes and beliefs |  |
| 43 | Rhondali, W., Burt, S. et al., 2013 | Hesitancy in initiating referrals (unless ‘sentinel’ events were present).  No clarity when stopping chemotherapy.  Misconception PC incompatible with cancer therapy.  No clarity about a clear-cut point when to initiate a palliative approach.  Communication regarding the supportive care option was challenging (e.g. referral to the program may lead to conflicts).  Patient denial of their severe condition.  Conflicts regarding goals of care.  Concerns disrupting balance between physicians. | Renaming ‘palliative care’ to ‘supportive care’ (patients were more receptive to the second name). | Strategies should consider physician curriculum development concerning communication skills, including how to communicate with colleagues to facilitate concurrent care. Additionally, trainings for oncologists should include systematic rotation in the PC department, and role-playing prognostic discussions and PC referrals with patients. | Attitudes and beliefs | PC referral |
| 44 | Rodriguez, K. L., Barnato, A. E. et al., 2007 | Related to health care providers:  Lacking knowledge about the aims and benefits of PC among health care providers.  Beliefs about PC should be provided to terminal patients only.  Confusion about terms and targeted patients by PC.  Beliefs that PC incompatibility with hospital and emergency department saving life culture.  Beliefs that the hospital/ healthcare providers' reputation can be damaged due to PC provision.  PC change goals of care before they (the treating physician) are ready.  Physicians' concerns PC threats patient autonomy.  Patient/Family related:  Interpretation of earlier and broader PC consultations as a cost saving measure (rather than clinically beneficial measure). | Providing workforce development, education and training to HPs.  Improving financial reimbursement and sustainability for PC.  Fostering a hospital culture focused on the individual patient needs and goals. | Strategies to initiating timely PC consultations should consider to highlight the patient benefits of PC and assurances that this care will not threaten provider autonomy. | Attitudes and beliefs  Awareness | Earlier use of PC |
| 45 | Ronaldson, S. and Devery, K., 2001 | Difficulty in addressing patient fear when ‘palliative care’ services are introduced.  Lack of ready access to information regarding PC.  Association of PC with imminent death.  Fear towards PC among patients. | n/a | Strategies to smoothly transition to PC services should consider preparing the patients for the transition process by improving the patient and carer access to PC information. Additionally, specific training on PC, including communication skills, should be provided to HP, in particular to nurses. | Collaboration and communication  Awareness  Attitudes and beliefs  Emotions | Transition to PC |
| 46 | Rugno, C. P., Rebeiro Paiva, B. S. et al., 2014 | Barriers among patients:  Lack of understanding of what PC consists (understanding PC as pain management or a place to die only).  Lack of understanding of the shift in treatment.  Lack of understanding of role of the PC team.  Differing perspectives about hope when discontinuation of active treatment was communicated. Perceptions regarding  hope were identified as overoptimistim vs hopelessness. | n/a | Strategies should take into consideration that early referral to PC is associated with a less painful therapeutic transition, based on more accurate knowledge of the importance of PC. | Awareness  Attitudes and beliefs | Palliative care access |
| 47 | Sanjo, M., Morita, T., 2018 | Inappropriate timing for receiving information about PC units (among 50% of patients).  Information given at diagnosis for a small proportion of patients only (16%).  Not enough time to take the decision for patients and their family.  Not clear timing regarding patient admission to PC unit.  None to consult about next steps of care.  Feeling difficulty in talking about parent's illness to young children.  No provision of information from oncologist (47% found information about PC somewhere else).  Inadequate amount of information provided by health care professionals (for 55% of patients).  The timing to receive information was late (for 30% of patients).  Lacking communication skills among HPs (communication methods are not always appropriate, these should be improved among oncologists).  Engagement of patients after the provision of information should be improved among oncologists.  No additional information are provided when choosing PC, especially home care.  Lack of information provided by HPs on PC home services,  Lack of information on staffing provided by HPs.  Lack of information on available treatment provided by HPs.  Lack of information on treatment expenses provided by HPs. | n/a | The strategies propose that medical staff should understand what  information families need and provide enough detail for patients in order for them to imagine how the time spent in a palliative care unit woul be like.  Additionally, physicians should endeavor to improve methods of communication and engagement after the information provision. | Collaboration and communication  Awareness  Attitudes and beliefs | Palliative care information provision |
| 48 | Schenker, Y., Crowley-Matoka, M. et al., 2014 | Misconception of PC as an alternative philosophy of care incompatible with cancer therapy.  Belief that providing PC is an integral part of the oncologist’s role.  Lack of knowledge about locally available services. | n/a | Effective strategies to subspecialty PC referrals should consider to raise awareness of the benefits of PC and to facilitate collaboration between oncologists and PC specialists. | Awareness  Attitudes and beliefs | Specialized PC referral |
| 49 | Smith, C. B., Nelson, J. E. et al., 2011 | Concerns that PC referral would alarm patients and families.  Concerns that discussions of unfavorable prognosis and of preferences for end-of-life care would be harmful to patients and families.  Patients preference to focus on curative rather than palliative therapies. | Beliefs that PC specialists have more time to discuss complex issues. | Strategies aiming to improve integration of PC as part of cancer management should include improving the offer of PC throughout the trajectory of illness, including patients with potentially curable disease. | Attitudes and beliefs | PC consultation referral |
| 50 | Walshe, C., Chew-Graham, C. et al., 2008 | Complex appraisal of the referral situation and subjectivity aspects of referral decision making: referrals are influenced by professionals’ perspectives about their work (e.g. attitudes towards PC).  Careful negotiation of PC services (not mandatory use of PC services). | n/a | Strategies should consider to encourage practitioners to be more explicit about their referral behaviors. In particular, they should encourage physicians to be more open to themselves, other professionals and patients about they made particular choices. Additionally, referral policies need to recognize the extent of influences on referral, and to explain in more detail local referral expectations. | Collaboration and communication  Attitudes and beliefs | PC referral |
| 51 | Ward, A. M., Agar, M. et al., 2009 | Reluctance for referral by patients or families.  Refusal to take referral for patients receiving anticancer treatment.  Physicians’ feeling of failure when referring to PC.  No clarity on PC initiation. | n/a | Strategies should consider promoting healthcare multidisciplinary links, mutual respect and understanding, and consistency in service provision. | Collaboration and communication  Attitudes and beliefs  Emotions | Specialized PC access |
| 52 | Zhang, Z. and Cheng, W.W., 2013 | n/a | Assessing psychological condition and treating mental disorders of patient.  Involving the family in patient care decisions.  Informing the family on the medical condition and the aims of PC. |  | Collaboration and communication  Emotions | PC access |
